# Expression of a chimeric OXR protein remodels rice architecture for enhanced grain yield by modulating redox and hormonal pathways

**DOI:** 10.1111/tpj.71213

**Published:** 2026-11-09

**Authors:** Becerra Agudelo Evelyn, Torti Pablo, Eusebi Delfina, Céccoli Gabriel, Campi Mabel, Welchen Elina

**Affiliations:** ^1^ Instituto de Agrobiotecnología del Litoral (CONICET‐UNL), Cátedra de Biología Celular y Molecular, Facultad de Bioquímica y Ciencias Biológicas Universidad Nacional del Litoral Santa Fe 3000 Argentina; ^2^ Instituto de Ciencias Agropecuarias del Litoral (ICiAgro Litoral, CONICET‐UNL), Cátedra de Fisiología Vegetal, Facultad de Ciencias Agrarias Universidad Nacional del Litoral Esperanza 3080 Argentina

**Keywords:** TLDc domain, rice, ABA, Brassinosteroids, growth‐defence, redox homeostasis

## Abstract

The growth–defence trade‐off limits crop biotechnology because stress adaptation typically reduces productivity. We engineered and characterised OXR‐Q12 (Q12), a mitochondrial chimeric protein combining the regulatory N‐terminus of AtOXR1 with the catalytic TLDc domain of AtOXR2. In *silico* analysis predicted higher stability for Q12 than for native orthologues. Constitutive Q12 expression in Arabidopsis and rice (*Oryza sativa*) increased biomass, remodelled stem and vascular architecture, and enhanced grain yield. OsQ12 rice plants showed an ideal erect canopy leaf phenotype, productive tillering and a robust root system. Physiologically, Q12 conferred salinity and drought tolerance by establishing a primed basal redox state with elevated non‐toxic H_2_O_2_ and an expanded glutathione content. Depleting this H_2_O_2_ pool with exogenous ascorbate selectively reverses seedling growth, salinity tolerance, and leaf erectness, indicating that this basal redox priming is a principal driver of these phenotypic adaptations. Mechanistically, our data support a model in which Q12 uncouples stress resilience from growth penalties by attenuating brassinosteroid (BR) signalling, downregulating biosynthesis (*OsBRD1*) and positive transcriptional regulators (*OsBZR1*, *OsDLT* and *OsILI1*), and differentially modulating the abscisic acid (ABA) response: the stress‐protection is kept, while the side that normally brakes growth is attenuated. Q12 lines exhibit partial resistance to ABA‐mediated growth and germination inhibition, yet display transcriptional hyperinduction of stress‐protective marker genes (*OsNCED3/4*, *OsDREB2A*) upon salinity exposure. This redox‐mediated signal may enable rapid activation of protective pathways without typical metabolic costs or growth reduction. Our results show that engineering modular OXR proteins can overcome traditional physiological trade‐offs, supporting the development of high‐yielding, resilient crops.

## Significance Statement

We engineered a chimeric OXR‐Q12 (Q12) protein that functions as a mitochondrial regulator, uncoupling stress resilience from growth penalties in rice. Through a proposed redox‐dependent modulation of ABA–BR crosstalk, Q12 increases biomass, achieves ideal erect‐leaf architecture, boosts grain yield under optimal conditions, and enhances seedling salt tolerance. This study provides a strategy for developing high‐yielding, climate‐resilient crops by establishing a primed redox state that sustains growth under both optimal and stress conditions.

## INTRODUCTION

Plants are sessile organisms that must complete their life cycle where they germinate. To adapt, they have developed unique mechanisms that enable rapid acclimation and stress response while maintaining a highly plastic post‐embryonic developmental programme (Yang & Mackenzie, 2019). As autotrophic organisms, plants rely on endosymbiotic organelles (chloroplasts and mitochondria) to regulate energy metabolism and meet cellular demands (Welchen & Gonzalez, 2021). Exposure to adverse conditions, such as drought, salinity or temperature extremes, can disrupt cellular homeostasis and cause accumulation of potentially damaging metabolic products, including reactive oxygen species (ROS) (Sewelam et al., 2016). While ROS are inherent to aerobic metabolism and function as key signalling molecules in development and stress responses, their overaccumulation can cause significant oxidative damage. Plants counteract this threat via a complex antioxidant defence network (Tripathy & Oelmüller, 2012). An effective strategy for improving crop resilience, therefore, depends on enhancing the plant's ability to finely regulate its redox state, preserving ROS signalling functions while preventing cellular damage (H. Huang et al., 2019). This equilibrated scenario underscores the importance of identifying regulatory proteins that can optimise this delicate balance.

A key protein family that serves as a conserved hub for stress mitigation is the oxidation resistance (OXR) family, first identified through functional screens for genes conferring protection against oxidative damage in humans (Volkert et al., 2000). Homologues of OXR proteins are highly conserved across all eukaryotes, from yeast to mammals, but are notably absent in prokaryotes, suggesting a specialised function that co‐evolved with eukaryotic cellular complexity and compartmentalisation (Durand et al., 2007; Elliott & Volkert, 2004; Finelli et al., 2015; Finelli & Oliver, 2017; Jaramillo‐Gutierrez et al., 2010). The defining characteristic of this family is a highly conserved C‐terminus protein motif, known as the TLDc domain (Tre2/Bub2/Cdc16, LysM domain, catalytic), comprising approximately 180 amino acids (aa) (Doerks et al., 2002; Durand et al., 2007). This domain has a unique globular structure unlike any other known protein motif, making its function challenging to determine. However, compelling evidence from studies in mammalian systems indicates that the TLDc domain is the essential functional unit responsible for the family's protective role. Protein isoforms that primarily contain the TLDc domain have been shown to fully rescue cells from oxidative stress‐induced phenotypes (Durand et al., 2007; Finelli et al., 2015, 2019). Nevertheless, the presence of multiple family members with distinct, non‐overlapping functions suggests that sequences outside the TLDc domain also play significant roles.

A primary mechanism of action of OXR proteins is the upstream regulation of the antioxidant response. Rather than acting as direct ROS scavengers, OXR proteins modulate the expression of key antioxidant enzymes. For instance, loss of OXR1 in mosquitoes and mice impairs the induction of glutathione peroxidase (GPX) and catalase (CAT) under stress, increasing sensitivity to H_2_O_2_ (Finelli et al., 2015; Finelli & Oliver, 2017). This regulatory function extends to protecting genome integrity, where OXR proteins prevent oxidative damage to nuclear and mitochondrial DNA and influence cell cycle checkpoints to allow DNA repair. Recent studies have begun to uncover a more direct molecular mechanism, revealing that OXR proteins in yeast and mammals physically interact with and regulate the assembly state of the vacuolar‐type H^+^‐ATPase (V‐ATPase) (Eaton et al., 2021; Klössel et al., 2024). Because the V‐ATPase is a master regulator of organellar pH, nutrient sensing and cellular energy homeostasis, OXR modulation could provide a unifying mechanism explaining the remarkably pleiotropic and potent effects of this protein family on cellular fitness and stress resilience. Recently, it was also demonstrated that OXR mammalian proteins interact with growth regulatory proteins in the mTOR pathway (Oot & Wilkens, 2024). Whether these physical interactions are conserved in the plant kingdom remains to be established.

The OXR gene family is also conserved in the plant kingdom, with *Arabidopsis thaliana* possessing six members that contain the canonical TLDc domain. The expression of these plant *AtOXR* genes is induced by various abiotic stressors, including heat, salinity and UV radiation, which supports their involvement in plant stress responses (Colombatti et al., 2019). Our earlier work characterised the plant OXR family, particularly AtOXR2 (At2g05590), showing that its constitutive overexpression in Arabidopsis enhances multiple agronomic traits. AtOXR2 transgenic plants display increased vegetative growth, biomass and stem and rosette size, supported by improved photosynthetic performance and reduced water loss compared with Wild‐type (WT) plants (Colombatti et al., 2019). In addition to these growth benefits under optimal conditions, AtOXR2‐overexpressing plants display broad‐spectrum tolerance to both abiotic and biotic stresses and show enhanced resistance to the pathogens *Pseudomonas syringae* (Mencia et al., 2020) and *Plasmodiophora brassicae* (Mencia et al., 2021). The heightened pathogen resistance is mechanistically linked to constitutively elevated levels of the defence hormone salicylic acid (SA) and a primed state of defence‐related gene expression. This combination of enhanced growth and primed defence challenges the typical trade‐off in plant biology (Huot et al., 2014). The ability of AtOXR2 to bypass this trade‐off suggests that it operates at a key regulatory node that co‐optimises resource allocation for both growth and defence, possibly by enhancing the overall cellular energy budget. To confirm the translational potential of this technology, we demonstrated that constitutive expression of the sunflower (*Helianthus annuus*) homologue, HaOXR2, in maize also improves plant fitness and stress tolerance, indicating that the beneficial functions of OXR proteins are conserved across the dicot‐monocot divide (Torti et al., 2020). On the other hand, the TLDc domain‐containing protein IbTLD from sweet potato (*Ipomoea batatas*) has been implicated in stress responses, regulating antioxidant defences following wounding and conferring salinity tolerance (Chen et al., 2022; Lin et al., 2012).

Rice (*Oryza sativa* L.) is a cornerstone of global food security, providing a primary source of calories for more than half of the world's population. However, its productivity is under constant threat from a range of abiotic stresses, with soil salinity emerging as a significant and escalating constraint on arable land worldwide (Formentin et al., 2018). Rice is particularly vulnerable to salinity, especially during the critical seedling and early vegetative stages, when high salt concentrations can severely inhibit growth and development, resulting in substantial yield losses (Sackey et al., 2025). Overcoming this trade‐off is therefore a paramount objective for agrobiotechnology (Takai, 2024).

Building upon our previous work with native OXR proteins and the established role of the TLDc domain as a key functional module, we have developed a novel protein engineering strategy that may potentially amplify these beneficial effects. We constructed a chimeric protein, designated OXR‐Q12 (Q12), by fusing the N‐terminal half of Arabidopsis AtOXR1 (At4g39870) with the C‐terminal, TLDc‐containing half of AtOXR2 (At2g05590). The central hypothesis of this study is that the engineered chimera, which combines domains from two different family members, may have a functionality that exceeds that of either native protein on its own. The main objective of this research is to characterise, at both the phenotypic and agronomic levels, transgenic lines of Arabidopsis and rice that consistently express the Q12 chimera. Translating discoveries from a dicot model, such as Arabidopsis, to a monocot crop like rice presents inherent challenges, as regulatory networks and protein interactomes can differ significantly between these evolutionarily distant clades (Yaschenko et al., 2025).

We evaluated key performance traits, including biomass accumulation, photosynthetic efficiency, seed production and tolerance to salinity and water deficits. By demonstrating the efficacy of this engineered protein in a staple monocot crop, this work aims to validate a new biotechnological strategy for developing the next generation of high‐yielding crops. These climate‐resilient crops can overcome the conventional constraints of the growth–defence trade‐off.

## RESULTS

### Generation, characterisation and analysis of the Q12 subcellular location

The OXR protein family is characterised by a C‐terminal TLDc domain, which is highly conserved across all eukaryotes (Doerks et al., 2002). Functional studies in organisms ranging from yeast to humans have consistently implicated this domain as the primary site responsible for the protein's antioxidant activity, suggesting that the TLDc domain is a modular functional unit (Blaise et al., 2012; Elliott & Volkert, 2004; Finelli et al., 2016; Finelli & Oliver, 2017; Volkert & Crowley, 2020).

To test the hypothesis that the TLDc domain of AtOXR2 is sufficient to confer its protective functions and can be transferred as an independent module, a protein engineering strategy was employed. The chimeric protein OXR‐Q12 (Q12) was designed and constructed using genetic engineering techniques. This chimera involves the genetic fusion of a cDNA fragment encoding the N‐terminal portion of AtOXR1 (At4g39870; aa 1–202) to a fragment encoding the C‐terminal region of AtOXR2 (At2g05590; aa 128–303). This C‐terminal portion of AtOXR2 encompasses the complete TLDc domain, plus seven aa to the end of the coding sequence (Figure 1A, see Figure S1 for protein sequences). By placing the AtOXR2 TLDc domain in a non‐native protein context, we sought to explore the domain's functional autonomy, testing whether it can act independently of its native N‐terminal partner, which is presumed to provide regulatory or localisation cues.

**Figure 1:**
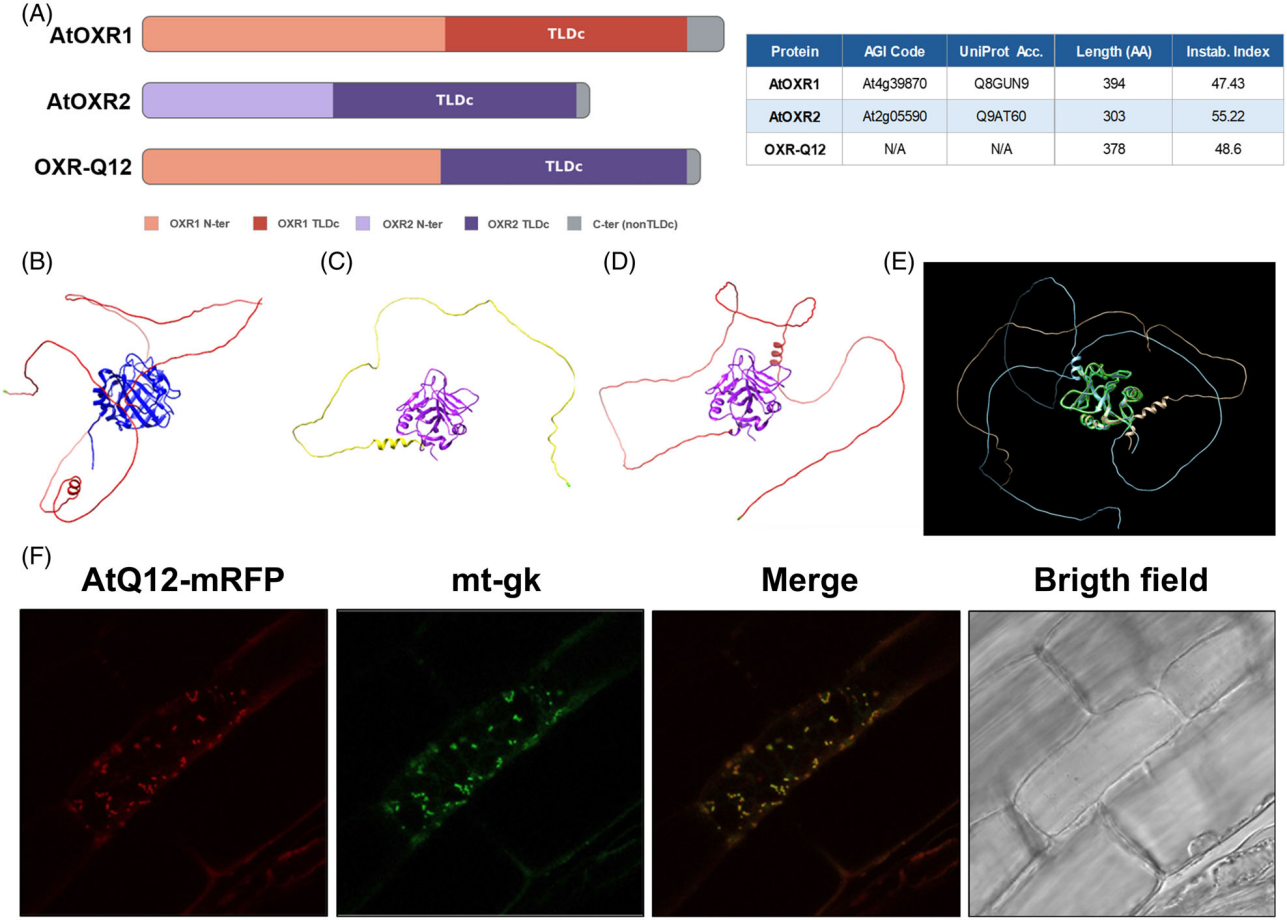
Design, *in silico* structural characterisation and subcellular localisation of the chimeric protein Q12. (A) Schematic representation of the domain architecture of *Arabidopsis thaliana* OXR proteins AtOXR1, AtOXR2, and the engineered chimera OXR‐Q12 (Q12). The N‐terminal domain of AtOXR1 (coral) was fused to the C‐terminal TLDc domain‐containing region of AtOXR2 (deep purple). The native N‐terminus of AtOXR2 is shown in light purple, and the native C‐terminus of AtOXR1, which also contains a TLDc domain, is in terracotta. The accompanying table provides Arabidopsis Gene Identifier (AGI) codes, protein size in amino acids (aa), UniProt accession numbers for the parental proteins, and the stability indices of the parental and chimeric OXR proteins. (B–D) Predicted tertiary structures of full‐length AtOXR1 (B), AtOXR2 (C) and Q12 (D), generated using the AlphaFold 3 algorithm (AF3) (Abramson et al., 2024). Mean pLDDT values: AtOXR1 = 68.69; AtOXR2 = 71.25; Q12 = 65.60. The models illustrate the structurally distinct N‐terminal regions and the conserved fold of the C‐terminal TLDc domain. (E) Structural superposition of the C‐terminal TLDc domains from AtOXR2 (cyan) and Q12 (green). Alignment and visualisation were performed with UCSF Chimera (Pettersen et al., 2004); RMSD (Cα) = 0.386 Å calculated over Cα atoms of residues 128–303 (AtOXR2) and 203–378 (Q12) (*n* = 165 aligned Cα pairs). The surrounding N‐terminal domains and unstructured regions are shown as thin traces for structural context. See Video S1 to explore the structure from different perspectives. (F) Subcellular localisation of Q12 in *Arabidopsis thaliana* root epidermal cells. The Q12 protein was C‐terminally fused to mRFP (Q12‐mRFP, red channel) and expressed in the mitochondrial marker line mt‐gk, which constitutively expresses GFP targeted to the mitochondria (mt‐gk, green channel). The Merge panel shows the overlay of the red and green channels, where the resulting yellow signal indicates definitive co‐localisation within mitochondria. The bright‐field image shows the corresponding root cells. Scale bar = 20 μm.

First, an *in silico* analysis was conducted to predict the structural integrity of the Q12 chimera protein. Data for the parental proteins were compiled using public repositories, including the Plant Proteomics Database (PPDB) (Sun et al., 2009) and UniProt (Ahmad et al., 2025). Key physicochemical parameters, including the instability index (II), which predicts protein behaviour and their relative stability in solution, were calculated using the ExPASy ProtParam tool (https://web.expasy.org/protparam/; Gasteiger et al., 2005). The parental proteins AtOXR1 and AtOXR2 had an II of 47.43 and 55.22, respectively. Generally, an II above 40 indicates that a protein is unstable, and the chimeric Q12 protein yielded an II of 48.6 (see Table in Figure 1A). This value suggests that the Q12 chimera is predicted to be less unstable in solution than its functional parent, AtOXR2. The lower predicted instability index suggests that the fusion is not only structurally tolerated but may be energetically favourable, providing a molecular starting point for subsequent functional studies *in vivo*. Additionally, we previously proposed that the four cysteines in the AtOXR2 TLDc domain are exposed on the protein surface accessible for redox reactions (Colombatti et al., 2019) (Figure S2). These four cysteines continue to adopt similar three‐dimensional conformations in the chimeric protein, suggesting that OXR‐Q12 could retain this putative function.

To evaluate the structural consequences of domain fusion, high‐confidence tertiary structure models were generated for the parental proteins AtOXR1 and AtOXR2, as well as for the chimeric Q12 protein, using the AlphaFold 3 prediction algorithm (Abramson et al., 2024). The resulting models revealed that, in all three proteins, the N‐ and C‐terminal regions fold into distinct domains (Figure 1B–D). The TLDc domain consistently adopts its characteristic compact globular fold, composed primarily of α‐helices interconnected by loops, irrespective of its N‐terminal fusion partner.

To quantitatively assess the structural fidelity of the engineered functional domain, the predicted structure of the Q12 TLDc domain was structurally aligned with that of the parental AtOXR2 model using the UCSF Chimera program (Pettersen et al., 2004). The alignment, which focused explicitly on the Cα atoms of the TLDc domain, yielded a root‐mean‐square deviation (RMSD) of 0.386 Å (Figure 1E; see Video S1). This exceptionally low RMSD value provides strong computational evidence that fusing the AtOXR1 N‐terminus does not induce significant conformational changes or perturbations in the native fold of the AtOXR2 TLDc domain. This structural preservation is a fundamental prerequisite for retaining biological function and aligns with the broader principle that protein domains often behave as independent, modular folding units (Lin & Liu, 2016; Reichmann et al., 2005).

Previous studies have shown that AtOXR2, as its homologue in yeast (Elliott & Volkert, 2004), is localised to mitochondria. To predict the localisation of the engineered chimera, the amino acid sequences of AtOXR1, AtOXR2 and Q12 were first analysed with the SignalP 6.0 server (Teufel et al., 2022). This analysis did not identify a canonical, cleavable N‐terminal signal peptide for mitochondrial targeting in any of the three protein sequences. To empirically determine the subcellular destination of the chimera, its coding sequence was fused in frame to the 3′ end of the gene encoding monomeric red fluorescent protein (mRFP), creating the Q12‐mRFP construct. This fusion construct was expressed in a stable Arabidopsis transgenic line, mt‐gk, which expresses GFP in mitochondria (Nelson et al., 2007). Confocal laser scanning microscopy of transformed root cells revealed that the red fluorescence signal from the Q12‐mRFP fusion protein was concentrated in discrete, punctate and often elongated structures that perfectly co‐localised with the green fluorescence signal from the mt‐gk mitochondrial marker (Figure 1F). The merged image unequivocally confirms that the Q12 chimeric protein is targeted to and accumulates within the mitochondria. The mitochondrial targeting of Q12 indicates that the signal is likely internal or conformational rather than a classical cleavable N‐terminus. Alternatively, the AtOXR1 N‐terminal region may itself provide mitochondrial targeting, as AtOXR1 also localises to mitochondria (data not shown).

### Constitutive expression of Q12 enhances vegetative growth, remodels plant architecture and boosts reproductive yield in Arabidopsis

To investigate the functional and phenotypic characteristics conferred by the chimeric protein Q12, we generated transgenic *Arabidopsis thaliana* lines that constitutively express Q12 under the control of the 35S CaMV promoter. Three independent AtQ12 homozygous lines, designated AtQ12‐1, AtQ12‐2 and AtQ12‐3, were selected (see Figure S3) for detailed phenotypic characterisation and were compared with WT and the previously described AtOXR2 overexpressor plant (Colombatti et al., 2019). Under greenhouse conditions, all AtQ12 lines exhibited enhanced vegetative growth relative to WT plants (Figure 2A) and an increased number of expanded leaves (Figure 2B). Quantification at 35 days after germination (DAG) confirmed a significant increase in rosette diameter (Figure 2C) and leaf number at bolting (Figure 2D). Moreover, the 10th rosette leaf exhibited a significantly larger area and higher dry biomass in AtQ12 plants compared with both WT and AtOXR2 controls (Figure 2E,F). Histological analysis of transverse sections at the base of the primary inflorescence stem revealed alterations in vascular development in the AtQ12 lines. Safranin–Fast Green staining revealed a markedly thicker, more intensely lignified vascular ring in the AtQ12 lines relative to WT plants (Figure 2G). Quantification confirmed that total stem cross‐sectional area was more than doubled in the AtQ12 lines (Figure 2H), while lignified secondary cell wall area increased by 3‐ to 4‐fold (Figure 2I), indicating a strong activation of secondary wall biosynthesis. Xylem analysis further revealed increases in both vessel number and vessel diameter (Figure 2J), resulting in a substantial expansion of total xylem area (Figure 2K). Collectively, these features indicate that AtQ12 plants develop a high‐capacity hydraulic architecture that may enhance water transport to support their enlarged leaf area efficiently.

**Figure 2:**
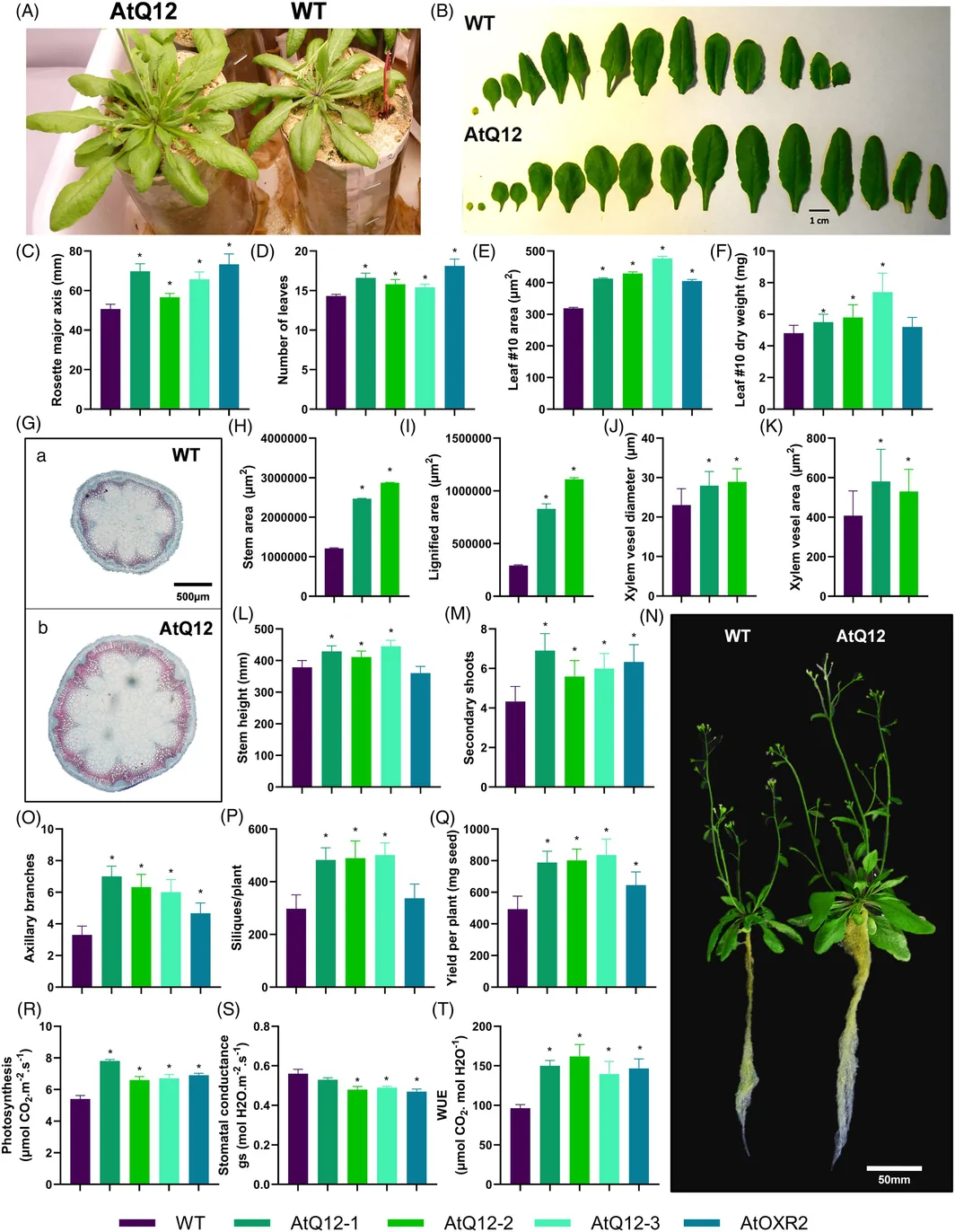
Phenotypic, anatomical and physiological characterisation of AtQ12 plants. (A) Representative image of 35‐day‐old AtQ12 and WT rosettes, (B) representative series of rosette leaves from mature AtQ12 and WT plants, arranged from oldest (left) to youngest (right), (C) rosette major axis length at 35 DAG, (D) total number of rosette leaves at bolting, (E) leaf area and (F) dry weight of the 10th rosette leaf. (G–K) Analysis of transverse sections of inflorescence stems: (G) Representative images of a transverse section at the base of the main stem from WT (a, top panel) and AtQ12 line (b, bottom panel) stained with Safranin–Fast green. Lignified secondary walls are stained adopting a reddish‐brownish colour, and primary cell walls are stained blue. Scale bar = 500 μm. (H) Stem cross‐section area measured from the base of the primary inflorescence stem, (I) lignified area within the stem cross‐section, (J) diameter and (K) area of xylem vessels per stem cross‐section. (L) Stem height measured at maturity, (M) number of secondary shoots per plant, (N) representative image of whole mature plants showing the architecture of AtQ12 vs WT. Scale bar = 50 mm. (O) Number of axillary branches per plant, (P) number of siliques per plant, (Q) total seed yield per plant. (R–T) Gas exchange analysis performed on mature rosette leaves: (R) Net photosynthetic rate (A), (S) stomatal conductance (gs), and (T) intrinsic water use efficiency (WUE), calculated as A/gs. For all bar graphs, data are presented as means ± SD. Asterisks (*) denote a statistically significant difference from the WT (Kitaake) at *P* < 0.05 (one‐way ANOVA with Dunnett's multiple comparisons test).

However, vegetative biomass is only agronomically relevant if it translates to reproductive yield. Thus, the substantial increase in vegetative biomass observed in AtQ12 plants prompted an investigation into their reproductive performance. Therefore, AtQ12 plants exhibited an increase in height relative to the WT and AtOXR2 plants (Figure 2L), but more importantly, a marked remodelling of inflorescence architecture. They produced significantly more secondary shoots (Figure 2M) and axillary branches (Figure 2O). This architectural change creates a scaffold capable of supporting a higher reproductive performance. The whole‐plant image visually confirms this, showing the AtQ12 plant as a multi‐branched structure compared with the slender WT (Figure 2N). These parameters translated into an increase in the number of siliques per plant (Figure 2P), resulting in higher total seed yield per plant in AtQ12 than in WT and AtOXR2 plants (Figure 2Q). Several growth parameters even surpassed those of the AtOXR2 control lines, indicating that Q12 optimises the source–sink relationship and promotes a more efficient allocation of leaf‐fixed carbon to seed production, thereby conferring superior overall plant performance compared with expression of the parental AtOXR2 protein.

Given the significant increase in biomass and yield in AtQ12 plants, we investigated key physiological parameters related to carbon assimilation and water use. Gas exchange measurements revealed that the net photosynthetic rate (A) was significantly higher across all three AtQ12 lines than in the WT (Figure 2R). In contrast, stomatal conductance (gs) was slightly lower in the AtQ12 lines (Figure 2S). The combination of increased carbon fixation and reduced or equivalent water loss resulted in a substantial improvement in intrinsic water use efficiency (WUE), calculated as A/gs (Figure 2T). These results indicate that Q12 not only promotes growth but also fundamentally improves the plant's ability to convert water into biomass.

### Constitutive expression of Q12 enhances seed yield under stress by mitigating oxidative damage in Arabidopsis

To evaluate the impact of Q12 expression on plant fitness and productivity under abiotic stress, the seed yield of WT and AtQ12 lines was quantified under water deficit and salinity‐imposed growth conditions. In response to water deficit, a significant reduction in seed yield was observed across all genotypes (Figure 3A,B). However, the AtQ12 lines consistently outperformed the WT and AtOXR2 plants under these conditions, maintaining yields that represented a substantial improvement in stress tolerance (Figure 3B). A similar trend of superior performance was observed under salinity (Figure 3C,D). Collectively, these results demonstrate that Q12 expression confers tolerance to abiotic stresses, consistently preserving plant reproductive success, even surpassing that of AtOXR2 plants.

**Figure 3:**
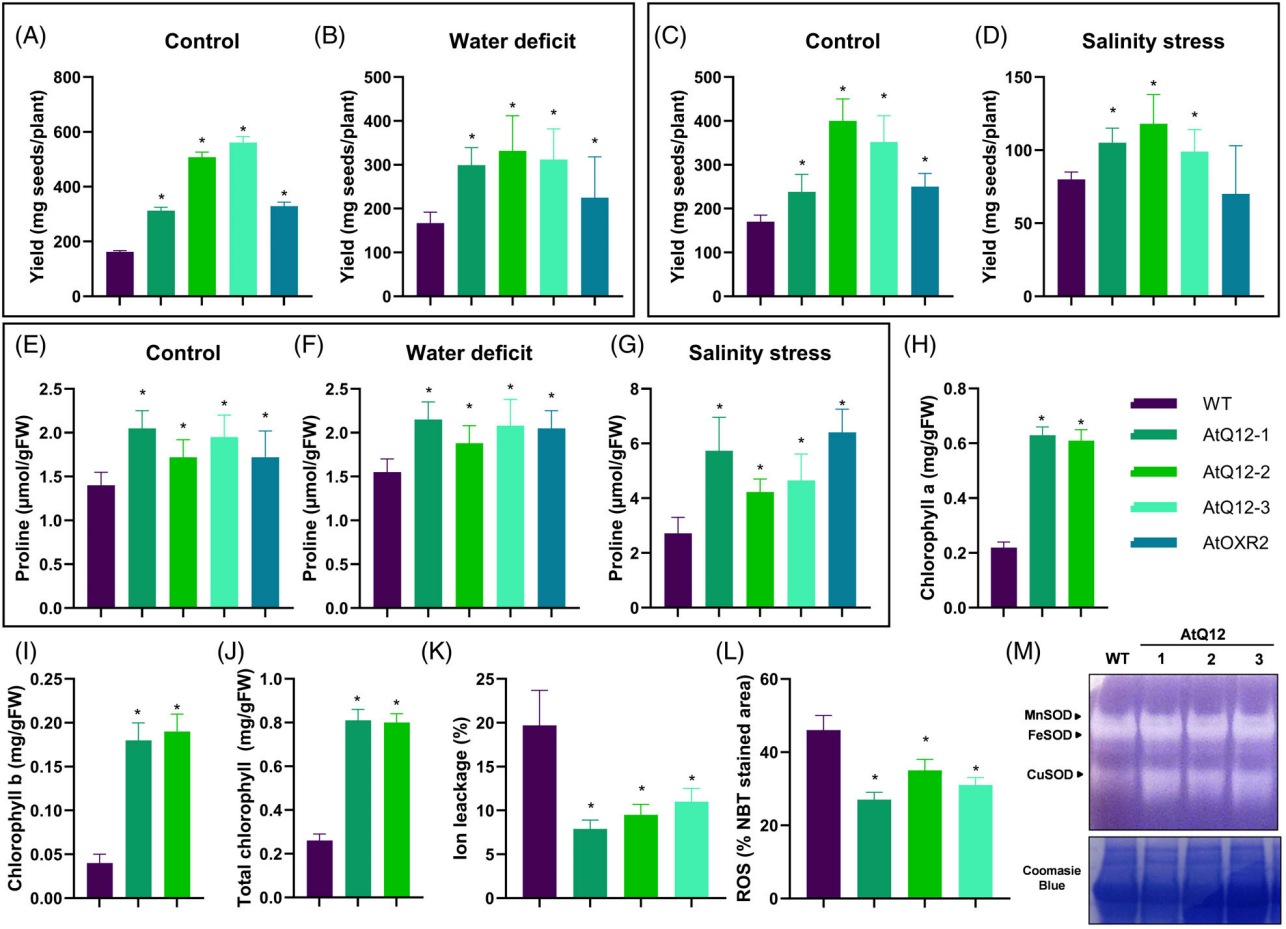
Constitutive expression of Q12 enhances stress tolerance and antioxidant capacity in *Arabidopsis thaliana*. (A–D) Seed yield of WT, AtQ12 lines (AtQ12‐1, AtQ12‐2, AtQ12‐3) and the AtOXR2 line grown under control conditions (A, C), or plant exposed to a water deficit (B) and salinity stress (150 mM NaCl) (D). (E–G) Free proline content in plants under (E) normal growth conditions (control), (F) water deficit and (G) salinity stress. (H–K) Physiological parameters measured in rosette leaves of WT and AtQ12 plants after salinity stress: (H–J) Photosynthetic pigment content measured as (H) chlorophyll a, (I) chlorophyll b, and (J) total chlorophyll content, expressed as mg/g fresh weight (FW). (K) Cell membrane stability assessed by ion leakage from leaf discs. Data are expressed as a percentage of total electrolytes released into the solution. (L) (%) NBT‐stained in foliar area of AtQ12 lines compared with WT plants. (M) Superoxide dismutase (SOD) activity assay in WT and AtQ12 plants under normal growth conditions: in‐gel activity image: Top panel: Native PAGE stained for SOD activity. Arrows indicate achromatic bands corresponding to putative SOD isoforms. Bottom panel: Coomassie Blue‐stained SDS‐PAGE showing equivalent total protein loading. For all bar graphs, data are presented as means ± SD. Asterisks (*) denote a statistically significant difference from the WT (Kitaake) at *P* < 0.05 (one‐way ANOVA with Dunnett's multiple comparisons test).

To investigate the physiological underpinnings of the observed stress tolerance, several biochemical parameters were assessed under stress. We therefore measured the free proline content in plants under control and stress conditions, with increased levels in AtQ12 lines (Figure 3E–G). Proline levels were elevated in AtQ12 plants under control conditions (Figure 3E). They remained significantly higher under water deficit (Figure 3F) and salinity stress (Figure 3G), supporting the view that AtQ12 lines exhibit a stronger osmoprotective response. Furthermore, under salinity stress, statistically significant differences were observed between the WT and the three AtQ12 lines in photosynthetic pigment levels, suggesting that Q12 expression improves the basal photosynthetic machinery (Figure 3H–J).

On the other hand, cell membrane integrity, a critical indicator of cellular health under stress (ElBasyoni et al., 2017), was evaluated by measuring ion leakage from leaf tissues. The AtQ12 lines exhibited significantly lower electrolyte leakage than WT plants after 7 days of salinity stress (Figure 3K), indicating enhanced membrane stability and increased oxidative protection. Since membrane damage is a hallmark of oxidative stress, we next quantified the *in situ* accumulation of superoxide (O_2_^−^) using NBT staining. Quantitative analysis of the stained leaf area revealed that all three AtQ12 lines accumulated significantly lower levels of superoxide, with stained regions ranging from 20 to 30%, in stark contrast to the WT (Figure 3L). To identify the mechanism responsible for the reduced superoxide levels, we performed an in‐gel activity assay for superoxide dismutase (SOD), the primary enzyme responsible for superoxide detoxification. The native gel stained for SOD activity revealed three distinct achromatic bands, corresponding to different SOD isoforms. Based on their electrophoretic mobility, these bands were putatively identified as manganese SOD (MnSOD, top band), iron SOD (FeSOD, middle band), and copper/zinc SOD (Cu/ZnSOD, bottom band). The AtQ12 lines exhibited markedly higher activity for the MnSOD and Cu/ZnSOD isoforms than WT (Figure 3M). These results demonstrate that Q12 expression specifically enhances the enzymatic antioxidant system. On the other hand, proline is a well‐known osmolyte that contributes to tolerance to drought stress (Renzetti et al., 2024).

### Expression of the Q12 chimera enhances biomass and modifies vascular architecture in rice

Before characterising the OsQ12 lines, we assessed whether the engineered Q12 chimera offers a structural rationale for its use in rice. We compared it with the closest native rice orthologue of the TLDc donor OsOXR2 (B9FWZ0), one of the six TLDc‐domain‐containing OXR proteins encoded in the rice genome. An *in silico* analysis analogous to that performed for the Arabidopsis parental proteins (Figure 1) showed that Q12 and OsOXR2 share a near‐identical TLDc fold: superposition of the AlphaFold 3‐predicted TLDc cores yielded a Cα RMSD of 0.477 Å (48.9% identity over the full‐length protein; 69.7% identity and 80.6% similarity over the TLDc domain) (Figure S4), comparable to the value obtained between Q12 and its parent AtOXR2 (0.386 Å; Figure 1E), whereas their N‐terminal regions were highly divergent. A domain‐resolved ProtParam analysis showed that full‐length Q12 is less unstable than native OsOXR2 (instability index (II) 48.6 versus 56.3). The difference comes from the N‐terminal region: the isolated TLDc domains had near‐threshold instability indices (Q12, 46.4 and OsOXR2, 39.4). The engineered OXR‐Q12 chimera, therefore, gives rice a TLDc catalytic module that is structurally and physicochemically equivalent to native OsOXR2, but set within a more stable overall protein. These structural and physicochemical inferences are computational predictions and were not tested experimentally here.

To further investigate the utility of the Q12 chimeric protein as a biotechnological tool for crop improvement, stable rice (*Oryza sativa* L. var. Kitaake) lines were generated. Rice OsQ12 plants were transformed with a construct expressing the Q12 chimera under the constitutive 35S CaMV promoter. As a result, several OsQ12 events with the artificial transgene of interest inserted into their genomes were obtained via callus regeneration and confirmed by PCR using specific oligonucleotides (Tables S1 and S2). Based on these expression data, three independent homozygous lines from the T3 generation, designated L1, L3 and L13, which exhibited transcript accumulation ranging from low to high (Figure S5), were selected for comprehensive phenotypic and physiological characterisation.

The impact of Q12 expression on rice growth was evaluated at two key developmental stages under controlled hydroponic conditions (see Figure S6). At the early seedling stage (6 DAG), all three transgenic lines exhibited a significantly more vigorous phenotype and a larger overall size (Figure 4A). Quantitative analysis confirmed that the total length of OsQ12 seedlings was considerably greater than that of the WT (Figure 4B), an increase attributable to a significant enhancement in both aerial part length (Figure 4C) and primary root length (Figure 4D).

**Figure 4:**
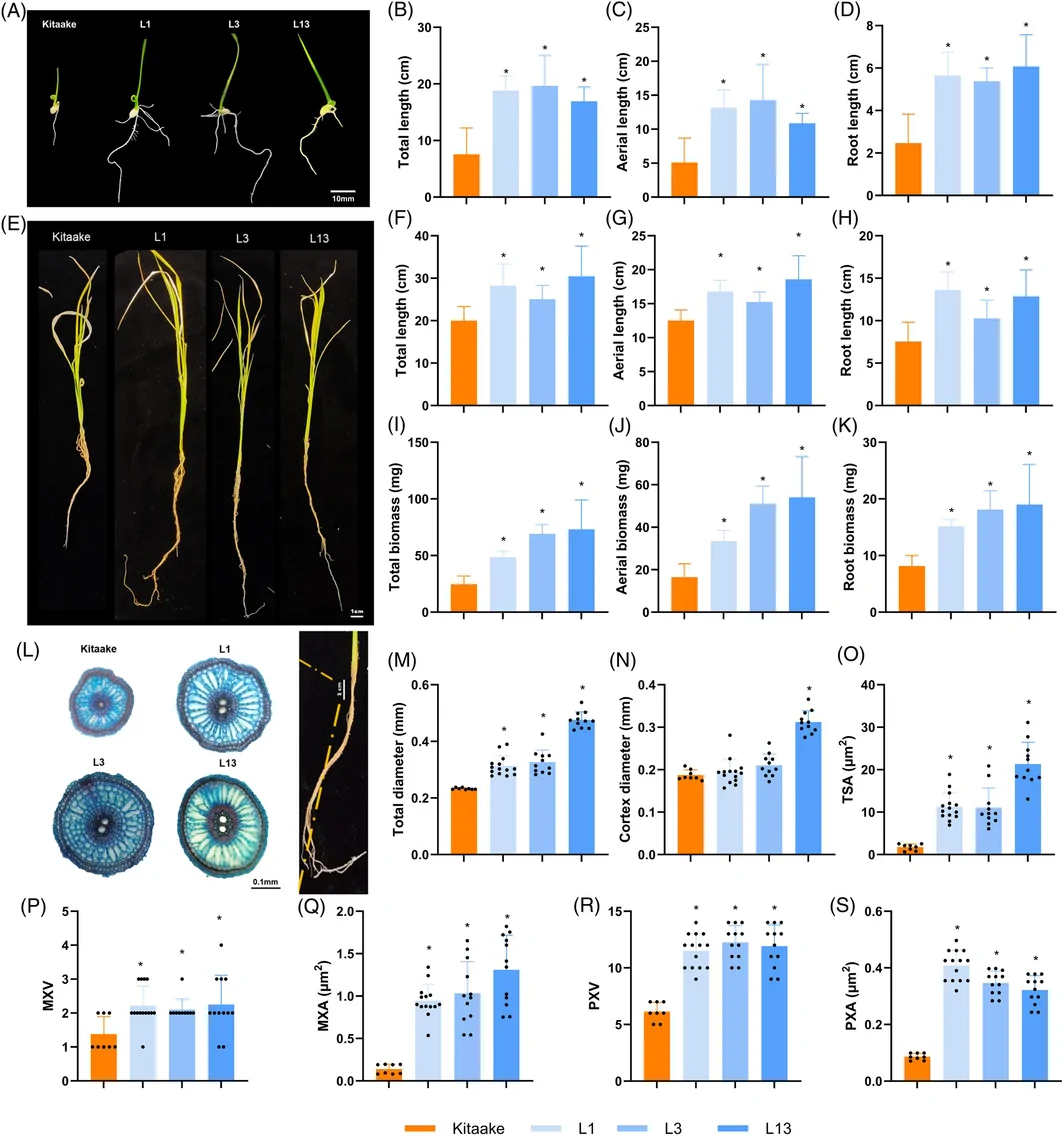
Expression of the chimeric protein Q12 promotes growth, biomass accumulation and enhances root vascular development in rice. Phenotypic and anatomical characterisation of WT (Kitaake) and three independent lines (L1, L3, L13) expressing the protein Q12 (OsQ12). (A–D) Analysis of 6‐day‐old seedlings grown hydroponically: (A) Representative images of seedlings from each line (Scale bar = 10 mm). Quantification of total seedling length (B), the aerial part length (C) and the primary root length (D). (E–K) Analysis of 30‐day‐old plants grown hydroponically: (E) Representative images of whole plants (Scale bar = 1 cm). Quantification of total plant length (F), aerial part length (G), total root length (H), total plant dry biomass (I), aerial part dry biomass (J) and root dry biomass (K). (L–S) Histological analysis of 30‐day‐old primary roots. (L) Representative transverse sections of primary roots stained with Toluidine blue. The inset image shows a representative root, with the dashed line indicating the location of the cross‐section (scale bar = 0.1 mm). Quantification of total root diameter (M), cortex diameter (N), total stele area (TSA) (O), number of metaxylem vessels (MXV) (P), total metaxylem vessel area (MXA) (Q), number of protoxylem vessels (PXV) (R), total protoxylem vessel area (PXA) (S). For all bar graphs, data are presented as means ± SD. Asterisks (*) denote a statistically significant difference from the WT (Kitaake) at *P* < 0.05 (one‐way ANOVA with Dunnett's multiple comparisons test).

This enhanced growth was maintained and amplified at a later vegetative stage. At 30 DAG, OsQ12 plants were visibly larger than WT controls (Figure 4E). Biometric parameter measurements confirmed that plant length (Figure 4F), aerial part length (Figure 4G), and root length (Figure 4H) were all significantly increased in the transgenic OsQ12 lines. This was directly correlated with increased biomass accumulation. The total dry biomass of 30‐day‐old transgenic plants was, on average, more than double that of the WT controls (Figure 4I). This increase was partitioned between both above‐ground and below‐ground tissues, with both aerial part biomass (Figure 4J) and root biomass (Figure 4K) showing statistically significant increases.

Given the significant impact on the root system, a detailed anatomical investigation was conducted on 30‐day‐old primary roots. Histological cross‐sections revealed profound structural changes in OsQ12 lines (Figure 4L). The overall diameter of the primary root was significantly increased in all three transgenic lines relative to the WT (Figure 4M). This enlargement was a composite effect of an expanded cortex diameter (Figure 4N) and a significant increase in the size of the central vascular cylinder, as shown by the significantly larger total stele area (TSA) (Figure 4O).

A comprehensive analysis of the vascular tissues reveals a marked increase in the plant's potential hydraulic capacity. The transgenic lines exhibit a higher number of large diameter metaxylem vessels (MXV) than the WT (Figure 4P), accompanied by a substantial increase in the total metaxylem vessel area (MXA) (Figure 4Q). Furthermore, the OsQ12 lines show a significant increase in the number of smaller diameter protoxylem vessels (PXV) (Figure 4R), leading to a notable increase in the total protoxylem vessel area (PXA) (Figure 4S). Collectively, these findings demonstrate that Q12 overexpression increased both shoot and root growth and enlarged the root vascular system in all three lines (Figure 4L–S).

### Expression of the chimeric protein Q12 increases tiller biomass and grain production in rice

We also characterised phenotypic parameters related to the reproductive structure and grain production of the OsQ12 lines at the end of their life cycle. Phenotypic characteristics of mature plants showed a more heavily tillered phenotype than Kitaake, as well as a greater number of harvested panicles in OsQ12 plants (Figure 5A). The final height of OsQ12 plants was statistically indistinguishable from that of the Kitaake line used as the control (Figure 5B). Similarly, no significant differences were observed in fundamental structural traits, including the number of internodes (Figure 5C), the diameter of the first tiller (Figure 5D), and the diameter of the first internode (Figure 5E). This indicates that Q12 expression does not induce deleterious pleiotropic effects, such as dwarfism or abnormal culm development. Likewise, flowering time and overall life cycle duration were unaffected, with all genotypes reaching maturity within the same period under greenhouse conditions. However, a significant effect was observed on the plant branching architecture. All three independent transgenic lines exhibited a highly significant increase in the number of productive tillers per plant compared with the WT (Figure 5F). This prolific tillering phenotype directly translated into a substantial increase in total aerial biomass in the OsQ12 lines (Figure 5G). The partitioning of this biomass was also altered, as evidenced by the tiller index (TI), defined as the ratio of tiller biomass to total aerial biomass (International Rice Research Institute (IRRI), 2002). The TI was significantly elevated in all transgenic lines (Figure 5H), demonstrating a specific and efficient reallocation of metabolic resources towards the development of productive shoots, a key determinant of yield potential in cereals.

**Figure 5:**
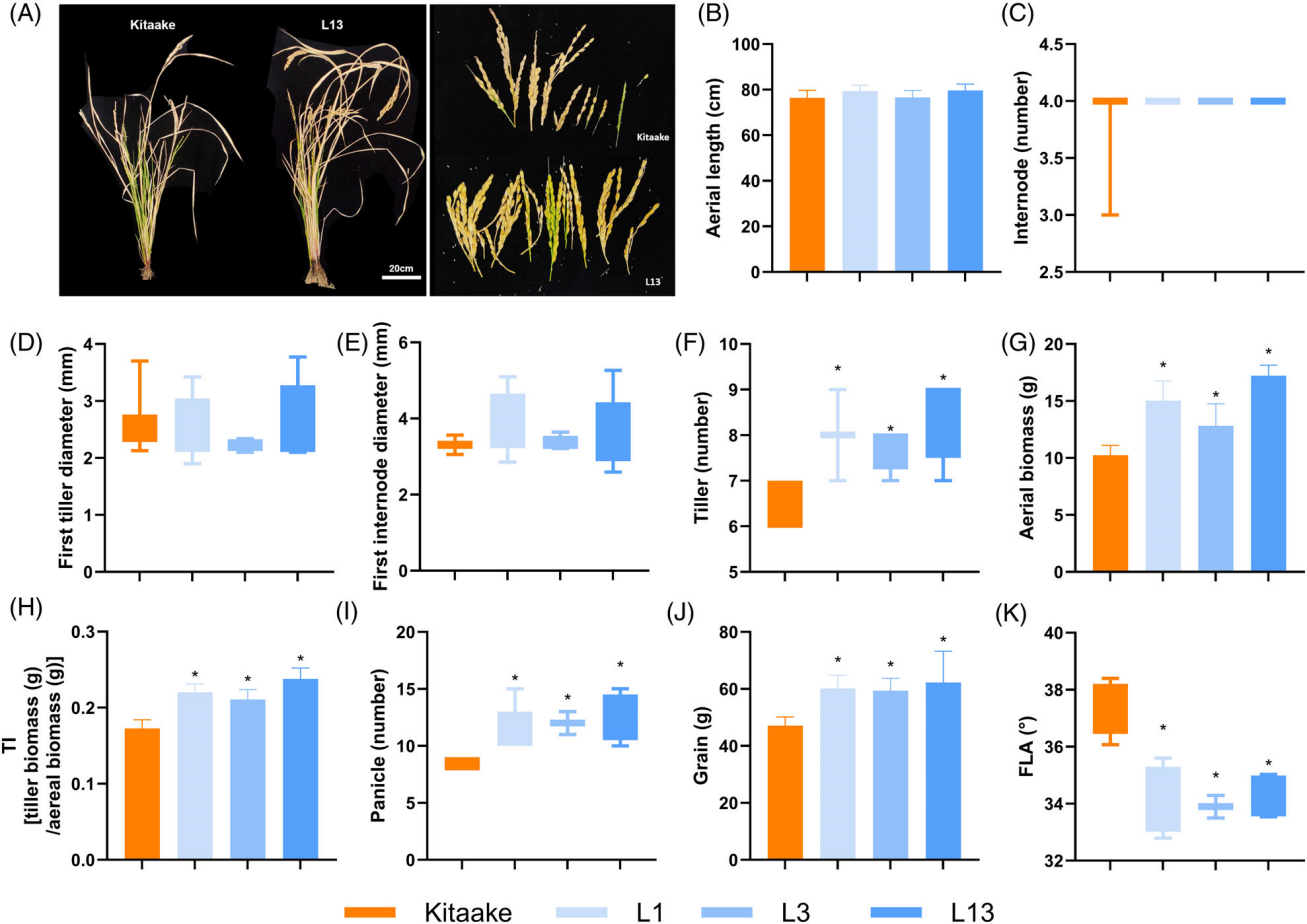
Expression of the chimeric protein Q12 enhances tiller biomass and grain yield in rice. Plants were grown under controlled greenhouse conditions for approximately 180 days until they reached maturity. (A) Representative images of mature Kitaake and L13 plants (left panel) and harvested panicles from a single representative plant of each genotype (right panel), scale bar = 20 cm. The following parameters were quantified on these plants: (B) aerial part length, (C) number of internodes, (D) diameter of the first tiller, (E) diameter of the first internode, (F) number of productive tillers per plant, (G) total aerial biomass, (H) tiller index (TI), calculated as the ratio of tiller biomass (g) to total aerial biomass (g), (I) number of panicles per plant, (J) total grain weight per plant and (K) flag leaf angle (FLA). For all bar graphs, data are presented as means ± SD. Asterisks (*) indicate a statistically significant difference compared with the Kitaake control as determined by a one‐way ANOVA followed by Dunnett's multiple comparisons test (*P* < 0.05).

Regarding parameters directly related to plant production, we quantified the primary components of grain production (Figure 5I–K). The substantial increase in tiller number observed in the transgenic lines led to a corresponding and statistically significant increase in the number of panicles per plant (Figure 5I). This result confirms that the additional tillers developed by OsQ12 plants are effective grain‐bearing shoots, a critical distinction for achieving higher yields (Yuan et al., 2024). Ultimately, this improvement in plant architecture culminated in a significant increase in overall grain yield (Figure 5J).

Additionally, we examined the flag leaf angle (FLA), another crucial trait associated with high yield potential. Our measurements revealed that all three OsQ12 lines displayed a statistically significant reduction in FLA compared with Kitaake plants (Figure 5K). The FLA is a key component of the ideal plant architecture concept in rice breeding, where more erect leaves allow better light penetration into the plant canopy, thereby improving overall photosynthetic efficiency and supporting higher planting densities, which is highly desirable for maximising grain production in an agricultural setting (Huang et al., 2021; Sakamoto et al., 2005).

### Expression of the chimeric protein Q12 modulates Brassinosteroid (BR) signalling, affecting the inclination of the lamina joint (LJ) angle in rice

Due to the positive impact of Q12 on rice production, the focus was placed on the LJ, the organ that connects the blade and the sheath, and is primarily responsible for determining leaf angle, a critical trait for plant architecture and photosynthetic efficiency and capacity (Gong et al., 2024; Sakamoto et al., 2005). Thus, a reduced LJ angle positively impacts photosynthetic capacity, thereby improving plant production (Li et al., 2020). The bending of the LJ is a direct result of differential cell proliferation and elongation, with greater elongation on the adaxial side relative to the abaxial side, leading to a larger leaf angle (Zhang et al., 2014, 2024) (Figure 6A). Quantitative analysis of the LJ angle of the second leaf in 8‐day‐old seedlings revealed a profound and consistent alteration in all three OsQ12 lines compared with Kitaake plants (Figure 6B,D). Thus, OsQ12 plants showed a reduced adaxial side length (Figure 6E) and an increased abaxial side length (Figure 6F), both of which contributed to lowering the LJ angle.

**Figure 6:**
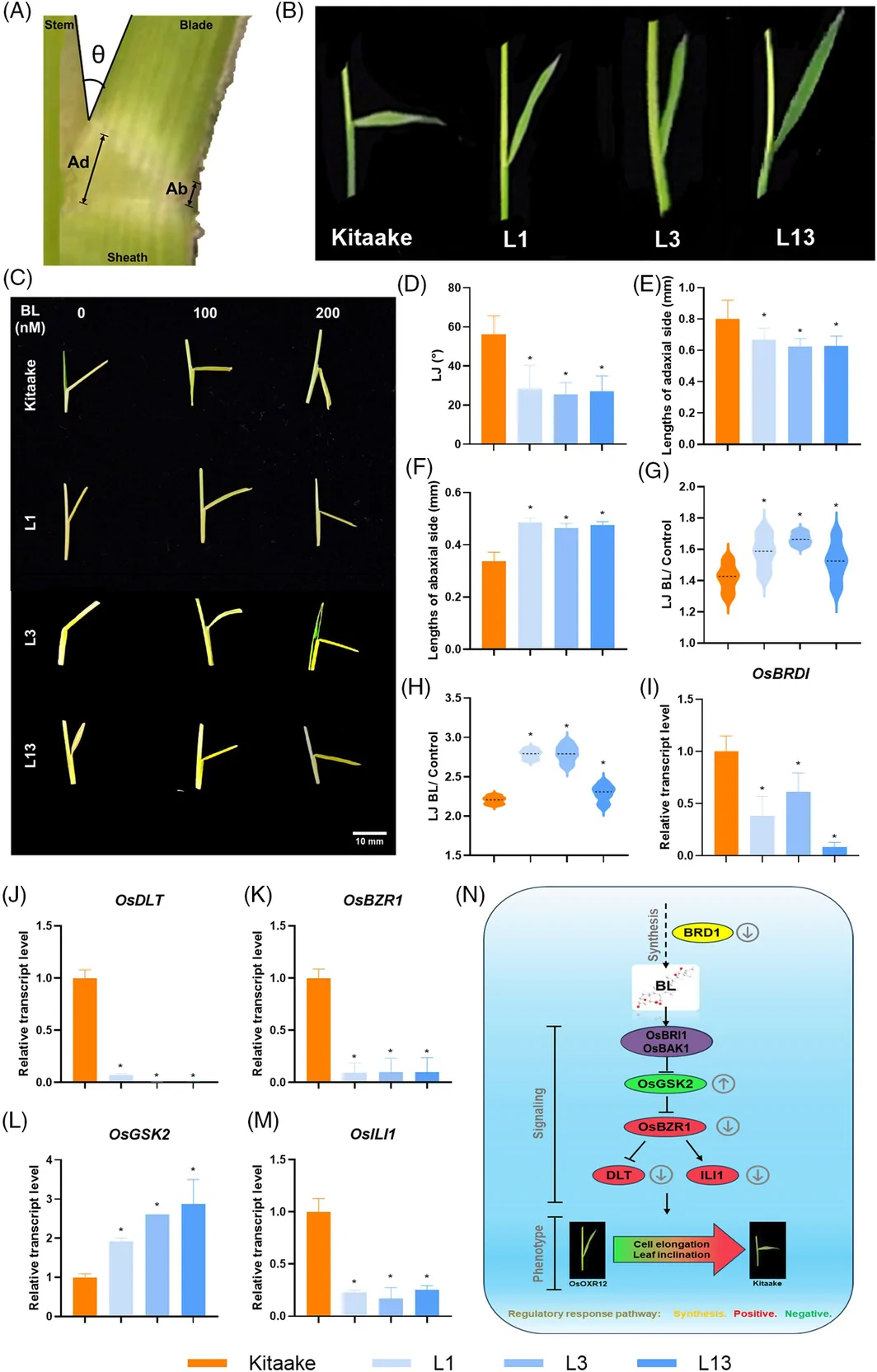
Constitutive expression of the chimeric protein Q12 induces an erect‐leaf phenotype in rice by attenuating the brassinosteroid pathway. (A) Schematic diagram of measured parameters at the lamina joint (LJ): inclination angle (θ), and the lengths of the adaxial (Ad) and abaxial (Ab) sides. (B) Representative images of the second‐leaf LJ phenotype in 8‐day‐old Kitaake, L1, L3 and L13 transgenic seedlings, illustrating the erect‐leaf posture in transgenic lines compared with the inclined leaf of the Kitaake. (C) Phenotypic characterisation and brassinolide (BL) response of OsQ12 lines: phenotypic rescue of the LJ angle in seedlings grown for 8 days on media supplemented with 0, 100 or 200 nM BL (scale bar = 10 mm). (D) Quantitative analysis of the second‐leaf LJ angle in 8‐day‐old Kitaake and three independent T3 homozygous OsQ12 lines. (E, F) Quantification of the adaxial (E) and abaxial (F) side lengths of the LJ. (G, H) Violin plots quantifying the BL response, expressed as the ratio of the LJ angle in BL‐treated versus mock‐treated seedlings (BL/Control) at (G) 100 nM and (H) 200 nM BL. (I–M) Relative transcript levels of key BR pathway genes in 8‐day‐old seedlings of Kitaake and OsQ12 lines, as determined by RT‐qPCR. Transcript levels were normalised to the *OsUBQ10* reference gene and are expressed relative to the Kitaake control. All bar plots show the mean ± SD of three independent biological replicates. Asterisks indicate a statistically significant difference from the Kitaake control (*P* < 0.05) as determined by one‐way ANOVA followed by Dunnett's *post hoc* test. (N) Simplified model of the BR regulatory pathway in rice controlling leaf inclination: BR biosynthesis leads to the active hormone (Brassinolide, BL), which is perceived by a cell‐surface receptor. This initiates a signalling cascade that inhibits the negative regulator *OsGSK2*, thereby activating the positive regulators *OsBZR1*, *OsDLT* and *OsILI1*, which promote cell elongation in the LJ and thus increase leaf inclination. Arrows indicate positive regulation or activation; T‐bars indicate negative regulation or inhibition. Genes analysed in this study are highlighted, and arrows indicate the observed transcriptional changes in transgenic lines.

Since the observed phenotype closely resembles that of plants impaired in BR biosynthesis or signalling (Li et al., 2019, 2021; Luo et al., 2016; Zhang et al., 2014), we hypothesised that the constitutive expression of Q12 may interfere with the BR regulatory network in rice. To test this, we used exogenous brassinolide (BL), the most active BR. We observed that the erect‐leaf phenotype of the OsQ12 plants was progressively rescued in a dose‐dependent manner, with the 100 nM BL treatment restoring the leaf angle to a WT‐like, inclined posture observed under normal growth conditions (see Figure 6C). We further assessed the sensitivity of the transgenic lines to BL. The violin plots illustrating the ratio of the LJ angle in BL‐treated versus mock‐treated seedlings (BL/Control) showed that all three OsQ12 lines responded significantly to exogenous BL application compared with the WT at both 100 nM BL (Figure 6G) and 200 nM BL (Figure 6H).

The heightened response to BL is characteristic of mutants with impaired BR biosynthesis and signalling, as they are effectively starved of the endogenous hormone. Thus, we performed a molecular analysis of key BR biosynthesis and signalling genes. We first examined *OsBRD1* (*BRASSINOSTEROID‐DEFICIENT DWARF1*) that encodes a C‐6 oxidase, a critical enzyme that catalyses a late step in BR synthesis (Hong et al., 2002). We found that *OsBRD1* transcript levels were significantly downregulated in OsQ12 lines compared with the WT (Figure 6I). We next analysed key components of the BR signalling cascade and found a coordinated response across the pathway. In all transgenic lines, *OsGSK2* (*GLYCOGEN SYNTHASE KINASE 2*), the rice orthologue of Arabidopsis AtBIN2 and a central negative regulator of BR signalling, was strongly upregulated (Figure 6J). At the same time, the expression of its downstream targets was markedly reduced. These included *OsBZR1* (*BRASSINAZOLE‐RESISTANT* 1), the master transcription factor executing BR responses (Figure 6K), and two positive regulators, *OsDLT* (*DWARF AND LOW‐TILLERING*) (Niu et al., 2022; Tong et al., 2009, 2012) and *OsILI1* (*INCREASED LEAF INCLINATION 1*) (Bai et al., 2007; Lu et al., 2024; ZL. Zhang, Shin, et al., 2009), both of which were almost completely suppressed in the transgenic lines (Figure 6L,M).

The data collectively indicate that Q12 overexpression results in a strong attenuation of the BR pathway (Figure 6N). This occurs through multiple mechanisms: it reduces hormone synthesis by repressing OsBRD1, disrupts the signalling cascade by increasing levels of the central inhibitor OsGSK2, and downregulates key positive regulators, including OsBZR1 and OsILI1. This comprehensive repression of the BR signalling pathway provides a molecular explanation for the erect‐leaf phenotype.

### Expression of Q12 drives transcriptional reprogramming and salt tolerance in rice

We also investigated the functional role of the chimeric protein Q12 in a monocot system under stress. To evaluate this, we assessed the performance of the rice line OsQ12 under salinity stress, an important abiotic factor that limits rice productivity, particularly at the vulnerable seedling stage (Sackey et al., 2025). Hydroponic assays revealed that when seedlings were germinated and grown for 6 days in a medium supplemented with 90 mM NaCl, the growth inhibition typically induced by salinity stress was significantly mitigated in the OsQ12 L1, L3 and L13 lines compared with Kitaake plants (Figure 7A–G). The root system showed the most pronounced differences, with OsQ12 lines exhibiting 50–70% longer roots than WT under salinity (Figure 7C), resulting in significantly greater total seedling length (Figure 7D). This improved growth translated into biomass accumulation, with shoot and root fresh weights more than doubling those of the WT under salt stress (Figure 7E–G). These findings suggest that constitutive Q12 expression confers a clear physiological advantage under moderate salinity, enabling rice seedlings to maintain growth and preserve cellular integrity. Because salinity stress typically causes oxidative damage and chlorophyll degradation, we quantified chlorophyll content as a proxy for stress tolerance. Although all plants exhibited some pigment loss, OsQ12 lines maintained significantly higher chlorophyll levels than WT (Figure 7H), highlighting their capacity to mitigate salt‐induced oxidative damage. To explore the antioxidant basis of this protection, we quantified anthocyanin accumulation, a class of flavonoids with potent ROS‐scavenging activity. Under salt stress, all three OsQ12 lines showed a 2‐ to 3‐fold increase in total anthocyanin content relative to WT (Figure 7I).

**Figure 7:**
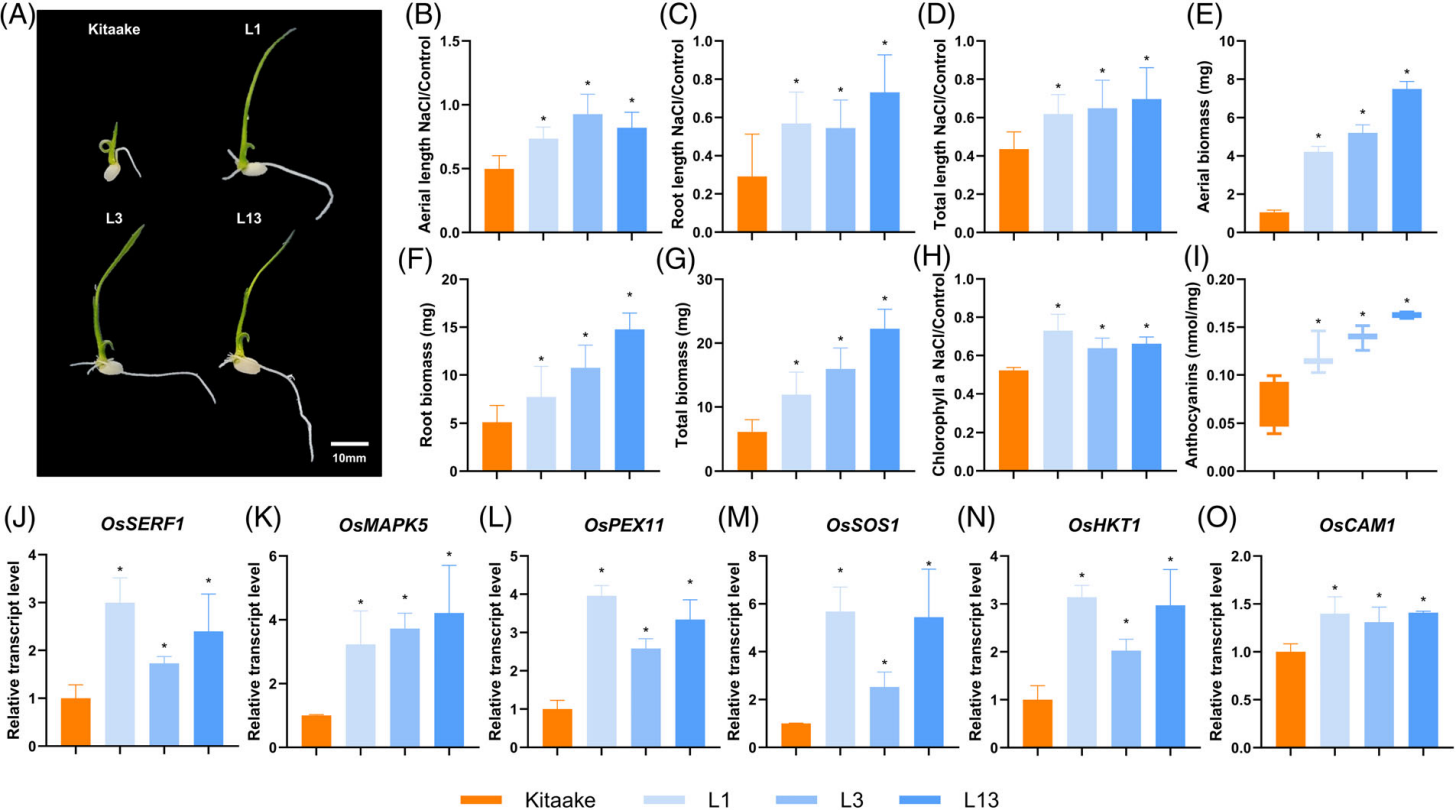
Expression of Q12 enhances salt tolerance in rice seedlings. (A–I) Phenotypic evaluation of seedlings grown hydroponically in the presence of 90 mM NaCl for 6 days. (A) Representative images of Kitaake and OsQ12 seedlings after 6‐day salt treatment. Scale bars = 10 mm. (B) Stem, (C) root and (D) total seedling length presented as a ratio of seedlings grown under 90 mM NaCl relative to control grown conditions (0.5X Hoagland). (E) Stem, (F) root and (G) total fresh biomass of seedlings grown under 90 mM NaCl. (H) Total chlorophyll content and (I) total anthocyanin content of seedlings presented as a ratio relative to NaCl to control grown conditions. (J–O) Molecular analysis of short‐term salt‐responsive gene expression. Eight‐day‐old seedlings grown in 0.5X MS liquid medium were exposed to 200 mM NaCl for 6 h. Relative transcript levels were determined by RT‐qPCR, normalised to the *OsUBQ10* housekeeping gene, and expressed relative to the Kitaake control plants under the same stress condition. All bar plots show the mean ± SD of three independent biological replicates. Asterisks (*) indicate *P* < 0.05 by one‐way ANOVA followed by Dunnett's *post hoc* test.

To dissect the molecular basis of the tolerance phenotype, we analysed the expression of key genes involved in salt stress signalling and ion homeostasis. Seedlings were exposed to an acute high‐salinity shock (200 mM NaCl for 6 h) to capture early transcriptional reprogramming events. Under these conditions, the OsQ12 lines mounted a rapid and coordinated response, with a significantly stronger induction of upstream regulators than WT. Transcript levels of *OsSERF1* (*SALT‐RESPONSIVE ERF 1*), an early‐response transcription factor that integrates ROS signals, translating the H_2_O_2_ signal into changes in gene expression (Schmidt et al., 2013), were markedly elevated (Figure 7J). Consistently, *OsMAPK5* (*MITOGEN‐ACTIVATED PROTEIN KINASE 5*), a positive regulator of salt, drought and cold tolerance (Xiong & Yang, 2003), was also upregulated (Figure 7K). The interplay between OsSERF1 and OsMAPK5 represents a critical regulatory node; OsSERF1 is required for the expression of salt‐responsive genes, while OsMAPK5 activity is positively correlated with abiotic stress tolerance (Schmidt et al., 2013). The concurrent activation of *OsSERF1* and *OsMAPK5* mirrors a known signalling module in which SERF1 directly induces *MAPK5*, thereby amplifying the stress response (Schmidt et al., 2013; Xiong & Yang, 2003). The activation of this signalling cascade was accompanied by the induction of downstream effector genes involved in oxidative protection and ion homeostasis. Transcript levels of *OsPEX11*, a peroxisomal biogenesis factor that promotes antioxidant activity and supports ion transporter expression (Cui et al., 2016), were significantly elevated in OsQ12 lines (Figure 7L). Its higher expression likely contributes to the superior ROS‐scavenging capacity required to maintain the stay‐green phenotype. Consistently, the structural genes for ion transport showed a coordinated upregulation. Expression of *OsSOS1* (*SALT OVERLY SENSITIVE 1*), encoding the plasma membrane Na^+^/H^+^ antiporter required for Na^+^ efflux (Martínez‐Atienza et al., 2007), was markedly upregulated (Figure 7M). In agreement, *OsHKT1* expression was significantly induced in the transgenic lines under salt stress (Figure 7N). OsHKT1‐family transporters function in retrieving Na^+^ from the xylem to prevent its accumulation in shoots (Khan et al., 2020). The induced expression of *OsHKT1* indicates a superior capacity for limiting Na + accumulation in the photosynthetically active mesophyll cells. Finally, our results show that *OsSOS1*, *OsHKT1;1*, and *OsCAM1* are upregulated synchronously in the OsQ12 lines. This triad represents the core of the Na + exclusion network. This was further supported by increased expression of *OsCaM1‐1*, a calmodulin gene that acts as a Ca^2+^ sensor, mediating ABA accumulation and limiting oxidative damage (Wei et al., 2025; Figure 7O).

### Molecular and physiological characterisation of Q12‐overexpressing rice: Uncoupling stress tolerance from growth penalties

To understand how Q12 influences rice physiology, we aimed to identify the mechanisms that allow OsQ12 rice plants to achieve both enhanced growth and increased salt tolerance. Specifically, we investigated whether these benefits are linked to changes in ABA homeostasis and sought to clarify the underlying molecular mechanisms. Therefore, we began by analysing the expression of key ABA biosynthesis and signalling genes in 9‐day‐old seedlings, both under normal growth conditions (Control) and after a short exposure to high‐salinity stress of 200 mM NaCl for 6 h (Stress) (Figure 8A–I). Under controlled conditions, the expression levels of *OsNCED3* and *OsNCED4*, which encode 9‐*cis*‐epoxycarotenoid dioxygenases implicated in ABA biosynthesis and salt stress tolerance (J. Li, Liu, et al., 2024; Xiang et al., 2024), were significantly reduced in OsQ12 lines (Figure 8A,B). Consistently, the expression of stress‐inducible signalling components, such as the markers *OsRab16A, OsDREB2A, OsPP2C49* and *OsbZIP23* (Ganguly et al., 2012; Kim et al., 2012; Singh et al., 2015), was also repressed relative to the Kitaake plants (Figure 8C,E–G). Two transcription factors typically repressed by ABA, OsAP2‐39, a key regulator of the ABA/Gibberellin balance in rice (Yaish et al., 2010), and OsWRKY24 (Li et al., 2023), were significantly downregulated in the OsQ12 lines under control conditions (Figure 8H,I). This coordinated repression is consistent with a primed, stress‐protective configuration of the ABA pathway, as we previously demonstrated for the homologous AtOXR2 protein in Arabidopsis (Colombatti et al., 2019). The ABA receptor gene *OsPYL/RCAR* exhibited elevated basal expression in the transgenic lines (Figure 8D), consistent with a sensitised, primed configuration of the protective ABA response.

**Figure 8:**
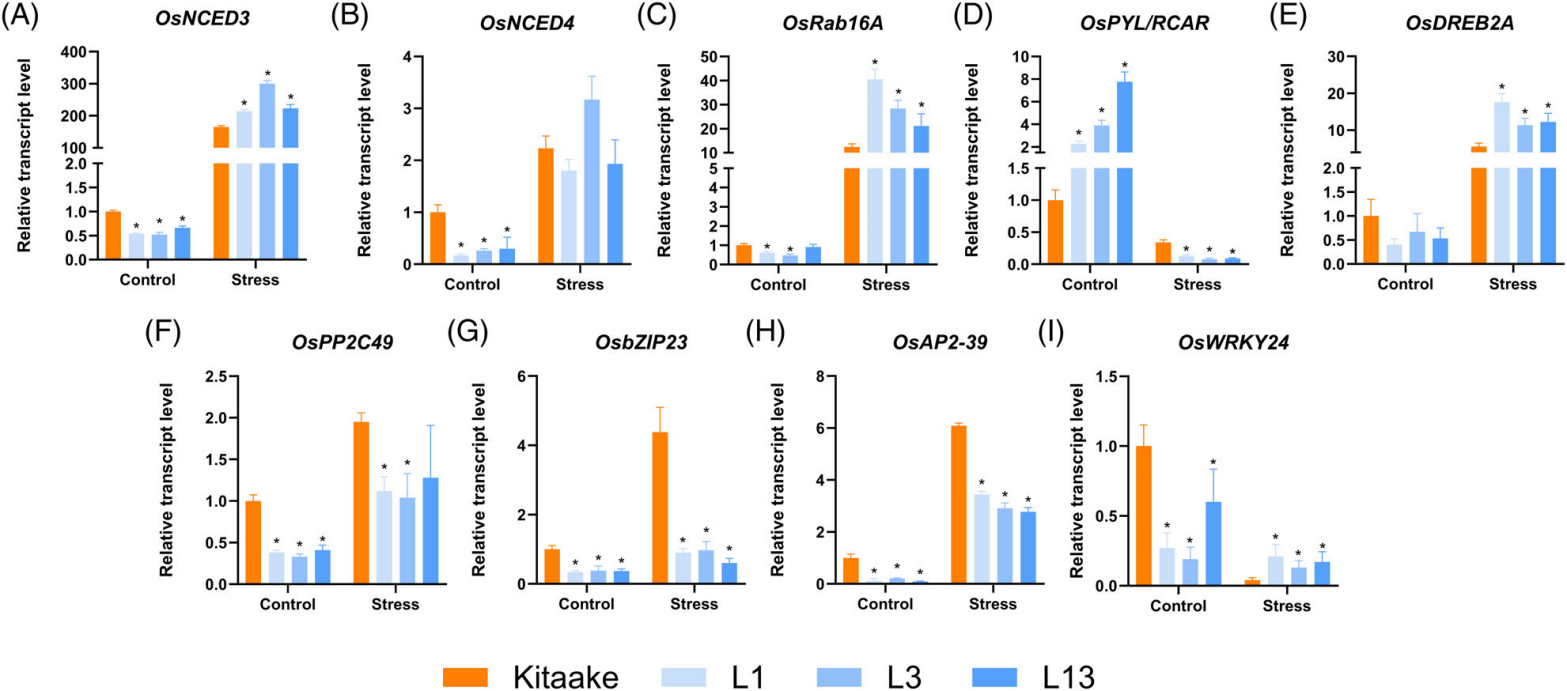
OsQ12 overexpression rewires ABA homeostasis and signalling. (A–I) Transcript levels of ABA biosynthesis and signalling genes in 9‐day‐old seedlings of WT (Kitaake) and three OsQ12 lines (L1, L3, L13), sampled under control conditions (0.5X MS) and after salt stress (200 mM NaCl, for 6 h): (A) *OsNCED3*, (B) *OsNCED4*, (C) *OsRab16A*, (D) *OsPYL/RCAR*, (E) *OsDREB2A*, (F) *OsPP2C49*, (G) *OsbZIP23*, (H) *OsAP2‐39* and (I) *OsWRKY24*. Transcript levels were normalised to *OsUBQ10* and the WT control set to 1. Bars show the mean ± SD of three independent biological replicates. Comparisons were performed by one‐way ANOVA followed by Dunnett's post‐hoc test, comparing each treatment (control and stress) to Kitaake within the same group. Asterisks (*) indicate *P* < 0.05.

Under imposed salinity stress, the OsQ12 plants displayed a significant hyper‐response in key defence‐related genes. While the WT plants showed the expected induction, the OsQ12 lines exhibited a stronger upregulation of *OsNCED3* and *OsNCED4* (Figure 8A,B). The expression of *OsNCED3*, the rate‐limiting enzyme in ABA biosynthesis, was induced to levels exceeding those in WT, indicating an enhanced ability to rapidly de novo produce ABA upon stress perception. The transgenic lines also showed higher expression of *OsRab16A and OsDREB2A* (Figure 8C,E), with OsDREB2A serving as a key integrator of phytohormone crosstalk and abiotic stress responses (Jang & Li, 2018; Zhang et al., 2013). In contrast, several components of ABA signalling showed the opposite trend. The receptor gene *OsPYL*/*RCAR*, the negative regulator *OsPP2C49* (Miao et al., 2024), and *OsAP2‐39* were all repressed in OsQ12 plants (Figure 8D,F,H), consistent with a more efficient, stress‐protective ABA response. The repression of *OsAP2‐39* is particularly relevant, as it suggests that OsQ12 plants achieve enhanced stress tolerance without the dwarfism typically associated with elevated *OsAP2‐39* expression. Additionally, *OsWRKY24* (Figure 8I), another ABA‐repressed negative regulator, remained suppressed in OsQ12 plants, consistent with an activated stress‐response state.

### OsQ12 rice plants resist the growth‐inhibitory action of ABA while retaining its protective signalling

To test whether transcriptional changes in ABA signalling translate into physiological changes, we performed ABA dose–response assays during seed germination and post‐germination establishment (Figure 9). Exogenous ABA markedly reduced the inhibition of OsQ12 lines compared with WT. Germination rates remained higher under 1 and 5 μM ABA (Figure 9A–C; Figure S7A,B). The same resistance appeared during seedling establishment. WT seedlings grown with ABA were shorter in both shoots and roots, whereas OsQ12 seedlings continued to grow (Figure 9B; absolute lengths in Figure S7C–E). Because the OsQ12 lines are already larger than the WT without ABA, we scaled each measurement to the genotype's own control. On that relative scale, the difference held: root length from the germination assay was strongly reduced by ABA in the WT but far less so in OsQ12 lines (Figure 9F), and shoot and root length in the establishment assay showed the same contrast (Figure 9G,H). The reduced inhibition is therefore specific to ABA rather than a by‐product of the larger baseline size. The LJ angle, expressed relative to the untreated control, was likewise reduced by ABA to a far lesser extent in OsQ12 plants than in WT (Figure 9I). Using a complementary approach, we performed the LJ inclination assay, a well‐established physiological readout of ABA sensitivity (Li et al., 2019), to evaluate the basal activation state of the ABA pathway in OsQ12 plants. This assay provided a phenotypic indicator for the interplay between growth and stress pathways, as LJ inclination reflects the antagonistic relationship between BR and ABA (Li et al., 2019; Trionfini et al., 2023). Consistent with the data presented above (Figure 6), OsQ12 plants exhibited a more upright LJ angle at baseline compared with WT (Figure 9J (left column, Control)). This phenotype is consistent with attenuated brassinosteroid signalling together with heightened responsiveness of the protective ABA branch, rather than elevated basal ABA content; both attenuated BR output and increased ABA responsiveness suppress cell expansion at the LJ and favour erect leaves (Li et al., 2019). Although OsQ12 plants also responded to ABA, their final LJ angle remained larger than that of WT (Figure 9J (+ABA), 9K). The attenuated straightening response suggests that OsQ12 plants are partially resistant to ABA, consistent with a shift in the BR–ABA balance.

**Figure 9:**
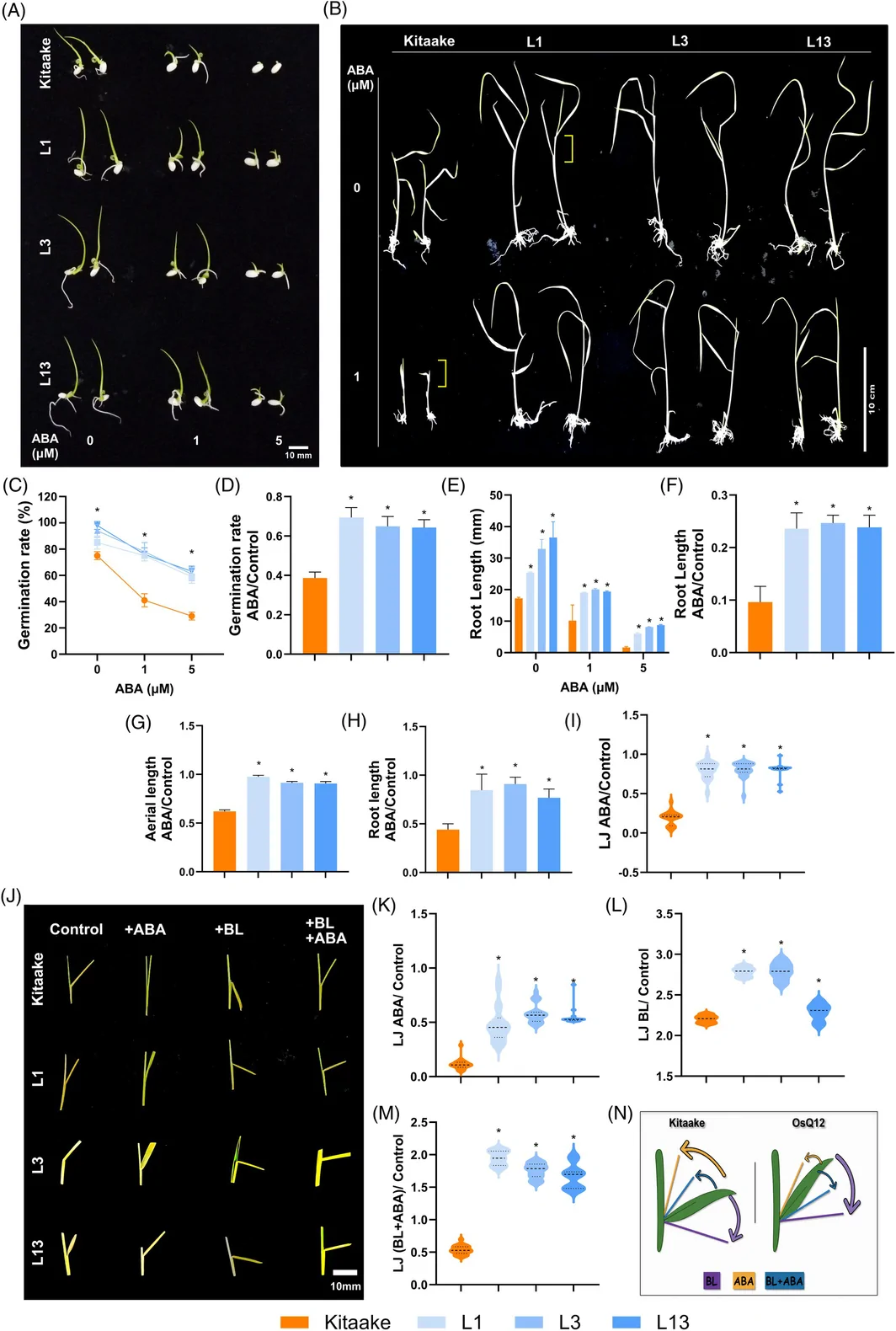
OsQ12 rice plants resist ABA‐mediated growth inhibition and re‐weight the BR–ABA antagonism. (A–H) ABA dose–response of WT (Kitaake) and OsQ12 lines (L1, L3, L13). (A) Representative seed germination of WT (Kitaake and three OsQ12 lines (L1, L3, L13) at 0, 1 and 5 μM ABA. Scale bar, 10 mm. (B) Representative post‐germination seedlings at 0 and 1 μM ABA; yellow brackets mark the segment considered for LJ evaluation. Scale bar, 10 cm. (C) Germination rate (%) at 0, 1 and 5 μM ABA, and (D) germination rate at 5 μM ABA relative to control, scored 4 days after sowing (DAS). (E) Primary root length at 0, 1 and 5 μM ABA and (F) root length at 5 μM ABA relative to control, scored 10 days DAS. (G) Shoot and (H) root length at 1 μM ABA relative to control (ABA/control). (I) Lamina joint angle relative to control (ABA/control), measured on the seedlings grown with ABA 1 μM, shown in (B). (J–N) LJ segment assay of ABA–BR crosstalk. (J) Representative second‐leaf LJ after 48 h in mock (control), 100 μM ABA, 200 nM brassinolide (BL) or BL + ABA (scale bar = 10 mm). Violin plots show the LJ angle as (K) ABA relative to control, (L) BL relative to control and (M) BL + ABA relative to control. (N) Schematic of the second‐leaf LJ response to BL, ABA and BL + ABA in WT and OsQ12. Graphs show the mean ± SD of three independent biological replicates (*n* = 30 seedlings per genotype and treatment). Asterisks indicate *P* < 0.05 (one‐way ANOVA, Dunnett's test against Kitaake).

In agreement, OsQ12 plants were hypersensitive to 200 nM BL, showing a significantly greater BL‐induced increase in LJ inclination than WT under basal conditions (Figure 9J (+BL), 9L), which aligns with the response observed in Figure 6. When LJ angles under the combined treatment (BL + ABA) were normalised to control conditions, OsQ12‐overexpressing lines showed a significantly higher response than Kitaake (Figure 9M), indicating that the inhibitory effect of ABA on LJ expansion was markedly reduced in OsQ12 plants. The BL signalling pathway overrides the ABA brake in OsQ12 plants, and ABA fails to antagonise the BL‐induced bending. Thus, BL led to a greater net increase in LJ inclination in OsQ12 plants under an ABA background. The schematic summary (Figure 9N) exhibits this differential response, showing that OsQ12 plants retain substantial BL‐induced leaf inclination under combined hormone treatment, whereas Kitaake exhibits a predominantly ABA‐restricted phenotype. Thus, Q12 overexpression attenuates ABA's inhibitory effect on BR‐mediated LJ expansion, allowing OsQ12 rice plants to sustain growth under conditions that would otherwise impose ABA‐dependent growth penalties.

Together, these assays provide direct physiological evidence that the growth inhibitory arm of ABA action is selectively attenuated in OsQ12 plants. In contrast, the stress‐protective ABA response is retained (Figure 8), mirroring the dual behaviour we previously reported for AtOXR2 in Arabidopsis, where seedling establishment was likewise less sensitive to ABA despite elevated endogenous ABA content (Colombatti et al., 2019).

### Q12 expression reprogrammes basal ROS homeostasis and orchestrates antioxidant defences

To investigate the mechanisms underlying enhanced salinity tolerance in OsQ12 lines, we hypothesised that stress resilience arises from a reprogramming of basal redox homeostasis, as observed in Arabidopsis plants overexpressing AtOXR2 (Mencia et al., 2020). To test this, we characterised basal ROS content and antioxidant activities in OsQ12 lines. Under normal growth conditions, the roots of 6‐day‐old OsQ12 seedlings showed markedly reduced dark blue formazan precipitates upon NBT staining, indicating lower superoxide radical levels than in WT (Figure 10B). Meanwhile, DAB staining showed the opposite trend, demonstrating a pronounced increase in basal H_2_O_2_ levels in OsQ12 plants (Figure 10C). In agreement, OsQ12 plants displayed higher total SOD activity (Figure 10D), consistent with reduced superoxide levels, while catalase activity was markedly reduced (Figure 10E). This opposing regulation suggests a coordinated remodelling of antioxidant enzymes, leading to an altered ROS profile in transgenic OsQ12 lines. The most pronounced change was a substantial expansion of the glutathione pool, with elevated GSH, GSSG, and total glutathione levels (Figure 10F), indicating a markedly enhanced redox buffering capacity even under non‐stress conditions. OsQ12 plants also displayed enhanced non‐enzymatic antioxidant defences, as evidenced by increased total phenolic and flavonoid contents (Figure 10G,H).

**Figure 10:**
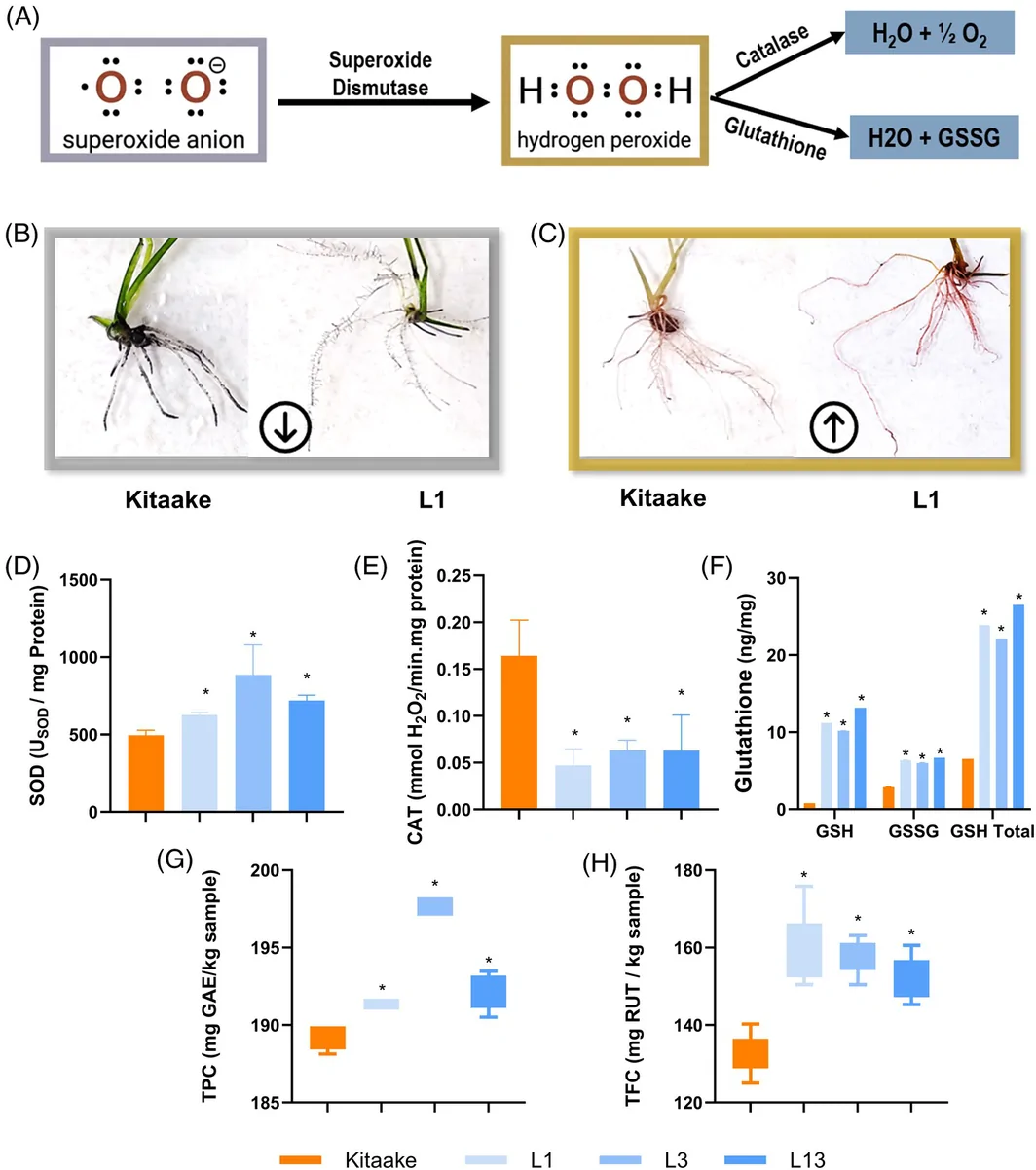
Overexpression of Q12 reprograms the basal antioxidant system in rice seedlings. Analysis was performed on 6‐day‐old WT (Kitaake) and transgenic OsQ12 rice seedlings grown under normal hydroponic conditions in 0.5X MS liquid medium. (A) Schematic representation of the ROS and enzymatic determinations performed in the plants. (B) *In situ* detection of superoxide anion (O2^·−^) in roots by nitroblue tetrazolium (NBT) staining. Dark blue formazan precipitates indicate the accumulation of O2^·−^. The downward arrow signifies a decrease in transgenic lines compared with WT (Kitaake). (C) *In situ* detection of hydrogen peroxide (H_2_O_2_) in roots by 3,3′‐Diaminobenzidine (DAB) staining. Intense reddish‐brown polymerisation indicates H_2_O_2_ accumulation. The upward arrow signifies an increase in transgenic lines. (D) Specific activity of superoxide dismutase (SOD), expressed as units per milligram of protein. (E) Specific activity of catalase (CAT), expressed as mmol of H_2_O_2_ decomposed per minute per milligram of protein. (F) Concentration of reduced (GSH), oxidised (GSSG), and total glutathione (GSH Total), expressed in ng/mg protein. (G) Total phenolic content (TPC), expressed as mg of gallic acid equivalents (GAE) per kg of sample. (H) Total flavonoid content (TFC), expressed as mg of rutin equivalents (RUT) per kg of sample. For all bar graphs, data are presented as the mean ± SD of three independent biological replicates. Asterisks (*) denote a statistically significant difference from the WT Kitaake at *P* < 0.05, as determined by one‐way ANOVA followed by Dunnett's *post hoc* test.

### Scavenging the basal H_2_O_2_ pool with ascorbate restores growth, salinity tolerance and leaf angle phenotypes in OsQ12 plants

The analysis above indicates that OsQ12 plants exhibit a higher basal H_2_O_2_ state, as evidenced by enhanced antioxidant capacity. Whether this redox state drives the improved performance of the transgenic lines or merely accompanies it cannot be determined by correlation. To separate the two, we supplied the growth medium with ascorbate (ascorbic acid, ASC), a membrane‐permeant reductant that lowers the cellular H_2_O_2_ pool (Smirnoff & Arnaud, 2019). If the phenotype depends on the elevated H_2_O_2_ pool, ascorbate should erode the advantage of OsQ12 plants.

Consistent with a depletion of the basal H_2_O_2_ pool, the DAB signal that was more intense in OsQ12 than in WT under control conditions was visibly attenuated in ascorbate‐treated OsQ12 roots (Figure 11A). More tellingly, ascorbate removed much of the growth advantage, and it is this quantified, genotype‐specific reversal (Figure 11B–J) that provides the causal evidence. Under both control and saline‐stress conditions, the longer aerial parts and roots of the OsQ12 lines were sharply reduced by ASC, whereas Kitaake plants, which start with little basal H_2_O_2_, were far less affected (Figure 11A,B,E). Under control medium, ascorbate had little effect on WT growth but shortened both shoot and root in the three OsQ12 lines. The (MS + ASC)/MS ratio remained close to unity in Kitaake and fell to roughly one‐half in L1, L3 and L13 for aerial length (Figure 11C,D) and for root length (Figure 11F,G). Ascorbate, therefore, removed most of the constitutive growth advantage of the transgenic lines. The salinity data pointed in the same direction. OsQ12 seedlings exhibited greater growth than the WT under NaCl (Figure S8A–C), consistent with their salt tolerance. Ascorbate reversed that relationship. In Kitaake, the treatment improved growth under salt, so the (NaCl + ASC)/NaCl ratio rose above one; in the OsQ12 lines, the same treatment lowered the ratio to about 0.45 (Figure 11E,H), and, compared with the unstressed control, the transgenic lines dropped below the WT once ascorbate was present (Figure S8D,E).

**Figure 11:**
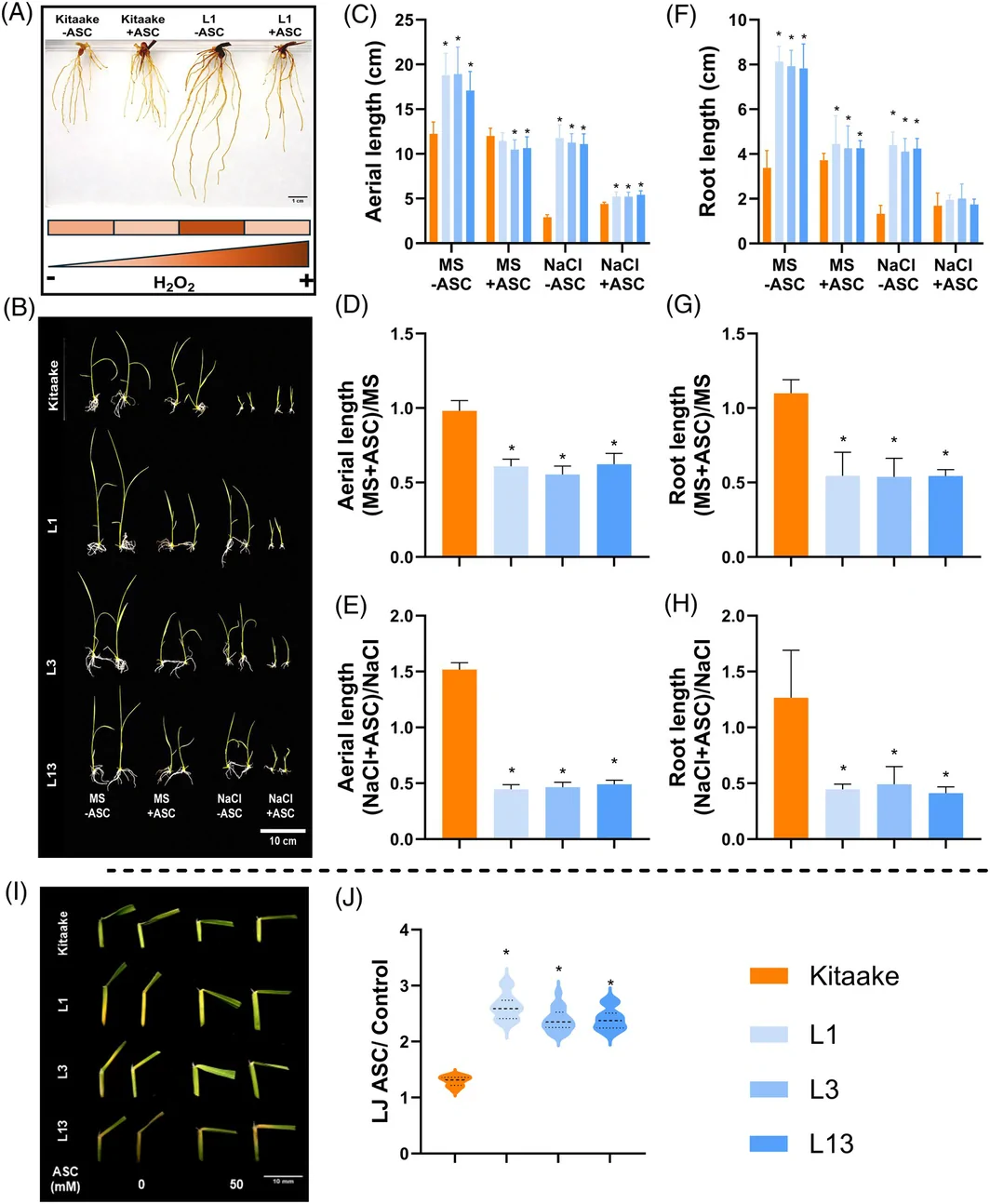
Scavenging H_2_O_2_ with ascorbate reverts the growth, salinity tolerance and leaf angle phenotypes of OsQ12 plants. WT (Kitaake) and three OsQ12 lines (L1, L3, L13) were grown on 0.5X MS medium without (−ASC) or with (+ASC) 5 mM ascorbate, under control or 120 mM NaCl conditions. (A) Representative roots of DAB‐stained Kitaake and L1 grown ±ASC; the bar below is a schematic gradient indicating the observed decrease in the H_2_O_2_ pool upon ASC addition (from + to −). Scale bar = 1 cm. (B) Representative phenotype of the Kitaake (top) and OsQ12 transgenic (L1, L3, L13) seedlings under MS (−ASC), MS (+ASC), NaCl (−ASC) and NaCl (+ASC). Scale bar = 10 cm. (C) Aerial and (F) root length under the four conditions. (D, G) Effect of ascorbate under control conditions, expressed as the (MS + ASC)/MS ratio for aerial and root length, respectively. (E, H) Effect of ascorbate under salinity, expressed as the (NaCl + ASC)/NaCl ratio. (I) Representative lamina joints of Kitaake and the OsQ12 lines after incubation with 0 or 50 mM ascorbate. Scale bar = 10 mm. (J) Lamina joint angle expressed as the ASC/control ratio. Data are shown as mean ± SD, *n* = 30 (bar graphs) and as violin plots with median and quartiles (J). Asterisks (*) indicate a significant difference from Kitaake within each condition at *P* < 0.05 (one‐way ANOVA followed by Dunnett's *post hoc* test).

Leaf architecture responded in the same manner. The erect‐leaf posture relaxed under ascorbate. The ASC/control ratio for the LJ angle reached about 2.5 at L1, L3 and L13, compared with 1.3 in Kitaake (Figure 11I,J). The upright leaves of OsQ12 plants are held in place by the same redox state and open when H_2_O_2_ is scavenged. Across three independent readouts, seedling growth, salinity tolerance and leaf angle, ascorbate moved the OsQ12 phenotype back towards the WT. This genotype‐specific reversion places the primed H_2_O_2_ pool upstream of the growth, tolerance and architectural traits. It provides the causal link between redox state and phenotype that correlational data could not provide on its own.

### Predicted functional networks of OXR2 recapitulate, *in planta*, associations previously inferred only from non‐plant systems

Due to the lack of experimentally validated protein–protein interactions for either OsOXR2 or AtOXR2 in IntAct or BioGRID, we utilised independent *in silico* resources to reconstruct their putative functional association networks (see Table S3). For OsOXR2, the highest‐confidence partners identified via FunCoup 5 (Persson et al., 2021) included two mitochondrial carriers, a Coenzyme A transporter (CoAc1; Q8S1A3), a Ca^2+/^metabolite carrier (Q5NAJ0), and a MAP kinase‐activating protein (OsMac3; Q75II8). Furthermore, STRING (Szklarczyk et al., 2023) retrieval associated OsOXR2 with the proton‐gradient regulator OsPGR5 and multiple subunits (A, B and D) of the vacuolar‐type H + ‐ATPase (V‐ATPase).

A parallel analysis of AtOXR2 yielded a highly complementary network. FunCoup recovered high‐confidence associates, including the TOR‐complex subunit LST8‐1 (Q9LV27), the protein phosphatase 2A regulatory subunit B'γ (Q8RW96) and GTG1 (Q9XIP7). Meanwhile, STRING identified the proton‐gradient regulator PGR5 and a coherent suite of chloroplastic NAD(P)H‐dehydrogenase (NDH) subunits (CRR3, PNSB2/3/4, PNSL1 and CRR23/NdhL). Interolog‐based structural modelling in PlaPPISite (Yang et al., 2020) revealed an association between AtOXR2 and the V‐ATPase subunit C (VHA‐C/DET3; At1g12840). Together with the V‐ATPase subunits retrieved for OsOXR2 by STRING, this indicates that the OXR–V‐ATPase association, previously described primarily in non‐plant systems, is independently recovered *in planta* for both homologues. The AtOXR2 interactant VHA‐C/DET3 is of particular interest because it functionally links the V‐ATPase proton pump to brassinosteroid signalling, guard‐cell Ca^2+^ responses, cell elongation and salinity tolerance (Allen et al., 2000; Dietz et al., 2001; Krebs et al., 2010; Schumacher et al., 1999) remarkably mirroring the physiological processes affected in our Q12/OXR2 lines.

Although the rice and Arabidopsis networks are not identical at the single‐gene level, they converge on the same core functional categories: (i) proton‐pumping and proton‐gradient regulation (V‐ATPase, PGR5, NDH); (ii) the TOR energy‐signalling axis (LST8); (iii) mitochondrial solute transport and (iv) redox‐ and hormone‐related signalling (PP2A‐B'γ, GTG1). Ultimately, the shared orthology of OsOXR2 and AtOXR2, combined with this functional convergence, strongly supports a conserved physiological context for OXR2 across monocots and dicots, providing hypotheses and a targeted roadmap for future experimental validation (Table S3).

## DISCUSSION

Rice (*Oryza sativa* L.) is a principal food crop for more than half of the world's population (Foley et al., 2011; Yuan et al., 2021; ICAR (Indian Institute of Millets Research Global Centre of Excellence on Millets), 2023). Global rice production is increasingly constrained by abiotic stressors, particularly salinity and drought, which disrupt cellular homeostasis and severely limit yield. A central paradigm in plant stress physiology is the growth–defence trade‐off, whereby activation of survival pathways, most notably those mediated by ABA, typically suppresses vegetative growth. This penalty arises from the antagonism between ABA and growth‐promoting hormones such as BR and GA. It explains why many stress‐tolerant transgenic plants exhibit dwarfism or reduced productivity, rendering such strategies unsuitable for agriculture (Zhai et al., 2025). This work identifies the Q12 chimeric protein as a biotechnology tool, with clear potential to engineer high‐biomass, high‐yielding rice varieties. The transgenic OsQ12 rice provides an opportunity to test whether this ‘biomass‐penalty‐free’ stress‐tolerance mechanism is conserved in monocots and to dissect the hormonal and redox networks underlying this phenotype.

The engineering of proteins with novel functionalities, such as Q12, was guided by the principle of protein domain modularity, which posits that individual structural domains can fold and function as autonomous units when repositioned in new molecular contexts (Jadwin et al., 2012; Lin & Liu, 2016). Here, we tested this principle within the Arabidopsis OXR family by creating Q12, a chimeric protein designed to isolate and functionally potentiate the TLDc domain of AtOXR2 by joining it to a divergent N‐terminal portion of AtOXR1. The *in silico* analysis predicted not only near‐perfect preservation of the native TLDc fold within the chimeric context but also a potential enhancement in overall protein stability compared with the parental AtOXR2 protein (Figure 1A,E). These predictions indicate that Q12 retains a well‐folded TLDc domain, a prerequisite for biological activity. Consistent with its design, Q12 localised correctly to mitochondria (Figure 1F), the primary site of OXR activity and the native compartment of both AtOXR2 (Colombatti et al., 2019) and AtOXR1 (not shown). Together, these results demonstrate that the AtOXR2 TLDc domain behaves as a portable, modular unit that maintains structural integrity and correct subcellular localisation even within a heterologous protein backbone.

This predicted stability advantage extends beyond the Arabidopsis parent to the native rice context: the closest rice orthologue of the TLDc donor, OsOXR2, shares a near‐identical TLDc fold with Q12 (69.7% identity, 80.6% similarity; Figure S4) yet, as a full‐length protein, is predicted to be more unstable than the chimera (instability index 56.3 versus 48.6). A domain‐resolved analysis localised this difference to the divergent N‐terminal regions rather than to the conserved catalytic core, whose isolated instability indices are comparable and near‐threshold (Q12 TLDc, 46.4; OsOXR2 TLDc, 39.4). This indicates that Q12 supplies a rice TLDc module that is functionally equivalent to the native protein and is present in a more stable overall protein context, rather than a more stable catalytic domain per se. Because these instability indices are sequence‐based heuristics dominated by the disordered, acidic N‐termini and because direct comparisons with OsOXR2‐overexpressing rice lines were not available, we regard this as a predictive, structure‐ and sequence‐level rationale that complements the experimental superiority of Q12 over its native parent AtOXR2 in Arabidopsis (Figures 2 and 3).

Collectively, our results demonstrate that Q12 is a potent positive regulator of organ growth, enhancing biomass (Figure 2). In Arabidopsis AtQ12 plants, we observed a pronounced increase in stem cross‐sectional area, driven by enhanced secondary growth and lignification (Figure 2G–K), which likely confers greater mechanical strength (Miedes et al., 2014; Voelker et al., 2010), while the expansion of xylem vessel diameter (Figure 2J) optimises hydraulic conductivity. This suggests that Q12 uncouples the typical trade‐off between safety and efficiency; it builds a stronger, more conductive stem, allowing AtQ12 plants to support their massive leaf area without experiencing water deficits even under stressful conditions (Figure 3), a key advantage over the native AtOXR2 phenotype. We also observed an increase in seed yield, which may be due not only to increased branching but also to a synergistic improvement in both resource acquisition and allocation. Larger leaves expand the photosynthetic source, increasing carbon assimilation, while enhanced axillary bud outgrowth produces a multi‐branched architecture capable of supporting a higher reproductive load (Figure 2L–Q). Underlying these morphological and yield gains is a marked improvement in photosynthetic performance. Q12 expression significantly increases CO_2_ assimilation per unit leaf area (Figure 2R), directly linking enhanced carbon fixation to greater leaf mass and whole‐plant productivity (Myat et al., 2022; Weraduwage et al., 2015). Similar results have been observed in plants overexpressing AtOXR2 (Colombatti et al., 2019). These photosynthetic improvements do not lead to increased water loss. Instead, AtQ12 lines exhibit a significant enhancement in WUE, achieving higher carbon assimilation while maintaining or even slightly reducing stomatal conductance (Figure 2S,T). This strategy enables greater biomass production per unit of water transpired (Bhaskara et al., 2022), in contrast to many approaches that improve WUE by restricting stomatal opening, often at the expense of CO_2_ uptake and growth (Blankenagel et al., 2018; Morison et al., 2008). By enhancing photosynthetic efficiency, Q12 bypasses this trade‐off, offering a promising strategy for boosting productivity and drought resilience (López‐Calcagno et al., 2020). This aligns well with biotechnological objectives aimed to optimise growth, photosynthesis and stress tolerance simultaneously (Islam et al., 2013). Our results demonstrated that the Q12 protein not only retained but also amplified the beneficial effects of AtOXR2. The growth enhancement observed in AtQ12 Arabidopsis lines, including a near threefold increase in rosette diameter and a substantial boost in seed yield (Figure 2), surpassed the phenotypes previously reported for the native AtOXR2 protein (Colombatti et al., 2019).

In parallel, the expression of Q12 in rice plants leads to a remarkable and agronomically favourable reprogramming of plant architecture, characterised by enhanced vegetative vigour and a profound expansion of the root vascular system (Figure 4). These findings position Q12 as a potent synthetic growth‐promoting protein and provide a compelling proof of concept for the use of engineered proteins in crop improvement (Leong et al., 2025). From the bottom up, a particularly striking feature in the roots of OsQ12 plants is the extensive remodelling of the vascular system. The marked increase in stele diameter, together with the proliferation and enlargement of metaxylem and protoxylem vessels (Figure 4L–S), reflects a substantial reinforcement of the plant's hydraulic infrastructure. This enhanced hydraulic capacity likely underpins the support of the larger shoot biomass and its higher transpirational demand, preventing water and nutrient supply from becoming a limiting factor for accelerated growth (Brodribb, 2009).

From the mitochondria, Q12 helps buffer the early ROS burst triggered by salinity stress, thereby maintaining a stable cellular redox state in both Arabidopsis and rice (Figures 3, 7 and 10). Thus, the pleiotropic benefits conferred by Q12 extend beyond simply growth promotion to include tolerance to principal abiotic stresses such as salinity and drought (Figures 3 and 7). This indicates the activation of a convergent stress‐protection pathway across monocots and dicots. The physiological basis for this enhanced resilience lies in a fundamental reprogramming of the plant's basal redox homeostasis, establishing a novel homeostatic state. Q12‐expressing plants displayed higher SOD activity (Figures 3 and 10). Strengthening the endogenous SOD system is a recognised strategy to increase stress resilience (Filiz et al., 2019; Kikuchi et al., 2015). Moreover, the enzymatic defence is complemented by elevated proline accumulation in Arabidopsis AtQ12 plants (Figure 3E–G). Proline plays a crucial role in osmotic adjustment, stabilising proteins and membranes and aiding in non‐enzymatic ROS scavenging (Renzetti et al., 2024). Together, these mechanisms provide a synergistic and stress‐protection system (Hayat et al., 2012). Additionally, Q12 expression strongly promotes anthocyanin biosynthesis, especially under salt stress (Figure 7I), further reinforcing antioxidant capacity and protecting the photosynthetic machinery from oxidative injury (G. Li, Guo, et al., 2024). These findings suggest that a primary function of the Q12‐mediated response is to potentiate the plant's antioxidant defence system, thereby protecting essential cellular components, such as chlorophyll and the photosynthetic machinery (Figures 3H–J and Figure 7H), from oxidative damage (G. Li, Guo, et al., 2024).

At the molecular level, our findings suggest that Q12 acts as a high‐level regulator, priming rice for a rapid, coordinated defence response under salt stress. Rather than activating a single pathway, Q12 overexpression amplifies an integrated stress‐response network. The most pronounced transcriptional differences between WT and OsQ12 plants occur within the OsSERF1‐OsMAPK5 module (Figure 7J–O). Given that OsMAPK5 interacts with and phosphorylates OsSERF1 (Schmidt et al., 2013; Xiong & Yang, 2003), its elevated expression in the OsQ12 plants suggests reinforced positive feedback loops that amplify ROS‐ and hormone‐derived signals. This upstream potentiation propagates downstream into multiple effector layers. One significant consequence is the strong induction of OsPEX11 (Figure 7L), which supports peroxisomal biogenesis, redox buffering and ROS‐scavenging capacity (Schmidt et al., 2013). This remodelling contributes to the superior antioxidant performance of OsQ12 plants and may underpin the observed stay‐green phenotype under salinity stress. A second outcome is the coordinated activation of an ion‐homeostasis module composed of OsCAM1, OsSOS1 and OsHKT1;1 (Figure 7M–O). OsCAM1 decodes early salt‐induced Ca^2+^ oscillations (Schmidt et al., 2013; Wei et al., 2025; Xiong & Yang, 2003). Its upregulation implies an enhanced capacity to translate Ca^2+^ signatures into transcriptional activation. Consistently, OsCaM1‐1 directly regulates the expression of OsSOS1 and OsHKT1;1 (Wei et al., 2025), aligning with their coordinated induction in OsQ12 plants. OsSOS1 increases Na^+^ extrusion at the plasma membrane (Martínez‐Atienza et al., 2007; Zhang et al., 2023). Collectively, these responses define a hierarchical stress‐response architecture in which Q12 enhances early Ca^2+^/ROS signalling, peroxisomal resilience and ion transport homeostasis. The concerted upregulation of OsSERF1‐OsMAPK5, OsPEX11, OsCAM1, OsSOS1 and OsHKT1;1 supports a model in which Q12 reinforces multiple regulatory layers rather than acting on a single downstream target.

OsQ12 rice plants display a remarkable combination of aerial traits, including increased tiller and panicle number, higher overall grain yield and a more erect canopy architecture resulting from reduced FLA (Figures 5 and 6). These traits closely align with the concept of ideal plant architecture pursued in modern rice breeding, designed to maximise light interception and photosynthetic efficiency under high‐density planting (Li et al., 2025; Zeng et al., 2017). The coordinated improvement of these diverse traits suggests that Q12 expression can overcome the trade‐offs that traditionally constrain yield optimisation (Takai, 2024).

Mechanistically, this erect‐leaf phenotype is underpinned by a profound alteration of the negative crosstalk between BR and ABA hormonal pathways (Y. Zhang, Bai, et al., 2009; Li et al., 2019). We demonstrate that the chimeric protein Q12 acts as a potent negative regulator of the BR signalling pathway, repressing both BR biosynthesis and signalling genes, while upregulating the key BR‐inactivating kinase OsGSK2 (Figure 6). This coordinated modulation prevents differential cell elongation in the LJ, which commonly causes leaves to bend (Hirano et al., 2017; Hughes, 2020; Qiu et al., 2025; Tong et al., 2012). A critical node in this crosstalk is the transcription factor *OsDLT*, identified by Li et al. (2021) as an integration hub downregulated by ABA. In our OsQ12 lines, *OsDLT* expression is virtually silenced (Figure 6). We propose that by depleting this key integrator, OsQ12 removes the BR pathway's buffering capacity, thereby locking the plant into an erect, stress‐avoidance architecture regardless of BR availability. The hormone‐induction assay shows this directly. In Kitaake plants, the two hormones act antagonistically at the LJ, and ABA holds the upper hand: applied together, ABA cancels the BL response and the joint stays closed (Figure 9). OsQ12 reverses that hierarchy. Brassinosteroids now prevail, the joint opens even in the presence of ABA (Figure 9L–N), and the plant reads a growth signal, whereas the WT reads a stop signal.

This reduced basal LJ angle is therefore primarily a consequence of BR pathway attenuation rather than of a generalised increase in ABA action. Although components of the ABA pathway are transcriptionally adjusted in OsQ12 plants, like elevated *OsPYL*/*RCAR* receptor expression and repression of the negative regulators *OsAP2‐39* and *OsWRKY24* (Li et al., 2017; Yaish et al., 2010; ZL. Zhang, Shin, et al., 2009; Zhao et al., 2020), this reconfiguration primes the stress‐protective branch of ABA signalling without enhancing its growth‐inhibitory action. Indeed, OsQ12 plants are markedly less sensitive than WT to the growth‐inhibitory effects of applied ABA on LJ expansion, germination and seedling establishment (Figure 9), reflecting a shifted BR–ABA equilibrium in which the growth‐cost arm of ABA is selectively attenuated (Li et al., 2019). In agreement with AtOXR2 plants (Colombatti et al., 2019), OsQ12 rice plants accumulate elevated yet non‐toxic basal levels of H_2_O_2_ (Figure 10C), a condition that reflects a constitutively primed state (Hossain et al., 2015; Saxena et al., 2016). In OsQ12 plants, this state arises from a coordinated enzymatic adjustment, in which increased SOD activity promotes H_2_O_2_ production. In contrast, reduced CAT activity limits its rapid detoxification, allowing H_2_O_2_ to persist at moderate concentrations, likely enabling it to function as a master regulatory signal rather than a damaging by‐product (Qureshi et al., 2022). This sustained H_2_O_2_ signal likely modulates stress‐responsive transcription and establishes stress memory, thereby enhancing preparedness for future oxidative challenges (Aswathi et al., 2025). The biochemical signature of this primed state includes a significant accumulation of phenolics and flavonoids, as well as an expanded glutathione pool (Figure 10F–H), strengthening redox buffering and ROS‐scavenging capacity. Together, these changes define a redox‐centred defence network that provides a mechanistic basis for the enhanced stress tolerance observed in OsQ12 lines.

The antioxidant analysis places OsQ12 plants in a more reducing, H_2_O_2_‐rich basal state, yet correlation alone cannot determine whether that state produces the phenotype or merely accompanies it. The ascorbate experiments settle the point. Supplying a reductant that lowers H_2_O_2_ stripped the transgenic lines of their extra growth, removed their salinity advantage and released the erect‐leaf posture. At the same time, the WT stayed unaffected or improved (Figure 11, Figure S8). The reversion was confined to genotypes that carry the primed redox state, which suggests that the elevated H_2_O_2_ pool lies upstream of growth, tolerance and architectural traits rather than downstream. Ascorbate is a general reductant, and its action is not restricted to H_2_O_2_, so we read the result as buffering of the primed redox pool rather than the loss of a single species; the agreement across three unrelated readouts, together with the opposite response of the WT, makes a non‐specific toxic effect unlikely. These data move the redox model from an association to a causal chain and set the mitochondrial H_2_O_2_ signal at its head, consistent with the expanded reduced glutathione buffering capacity described above.

At the molecular level, our data demonstrate that Q12 functions as a mitochondrial thermostat, recalibrating the plant's stress‐response threshold. By localising the TLDc domain to the mitochondria, Q12 modulates the quality of the ROS signal, in which elevated but non‐toxic basal H_2_O_2_ acts as the primary retrograde signal, while the expanded and more reduced glutathione pool buffers this signal and keeps it within a safe, non‐damaging range (Figure 10). This leads to a primed molecular signature that enables rapid stress‐induced activation of ABA‐dependent protective genes without imposing their full metabolic cost under non‐stress conditions (Kim et al., 2012, 2014). Crucially, this protective priming occurs alongside transcriptional quiescence of core biosynthetic genes under normal growth conditions (Figure 8), allowing metabolic energy to be allocated to biomass accumulation under favourable conditions, similar to mechanisms observed in Arabidopsis (Colombatti et al., 2019). This reprogramming also mitigates the typical growth–defence trade‐off. In WT rice, the AP2 transcription factor OsAP2‐39 links high ABA to GA catabolism, and its overexpression leads to severe dwarfism (Yaish et al., 2010). By contrast, salt‐stressed OsQ12 plants produce much less OsAP2‐39 than Kitaake Figure 8, indicating that they generate sufficient stress‐induced ABA (via OsNCED3) without triggering the excessive GA breakdown that would arrest growth. Likewise, OsWRKY24, a known negative regulator of ABA signalling (ZL. Zhang, Shin, et al., 2009), remains strongly repressed in OsQ12 lines, further de‐repressing ABA responses without incurring the usual growth penalty. These adjustments allow the transgenic plants to fortify their stress tolerance while maintaining biomass production.

Upon salinity stress, OsQ12 plants deploy an exceptionally efficient ABA response. Unlike WT, which exhibits a strategy involving massive induction of the activator OsbZIP23 concomitant with its inhibitor OsPP2C49 (Miao et al., 2024; Zong et al., 2016), OsQ12 lines show minimal induction of the phosphatase inhibitor. Consequently, downstream targets such as OsNCED3 and OsRab16A are hyperinduced, ensuring rapid osmotic adjustment and membrane protection (Usman et al., 2023). This signalling precision explains how OsQ12 plants escape the typical growth–defence trade‐off, in which constitutive ABA activity usually causes dwarfism (Feng et al., 2024; J. Li, Liu, et al., 2024; Wan & Li, 2006).

Q12 was expressed in rice under the constitutive CaMV 35S promoter. Monocot promoters such as maize *Ubi‐1*, rice *Act1*, or the rice ubiquitin promoters *RUBQ1/RUBQ2* generally give higher average expression in cereals (Christensen et al., 1992; McElroy et al., 1990; Wang & Oard, 2003), but the 35S promoter is active in rice leaf, vascular, and root tissue (Battraw & Hall, 1990) and here drove enough expression to produce consistent, dose‐dependent phenotypes in three independent homozygous T3 lines (Figure S5). Expression and phenotype were retained through the T3 generation, and we had already obtained 35S‐driven expression of the homologous protein HaOXR2 in maize (Torti et al., 2020); together, these indicate that promoter silencing did not compromise the lines studied here. Optimised monocot promoters would be a sensible next step for translational use.

In conclusion, Q12 functions as an upstream regulator that reprograms ABA signalling into a metabolically efficient, stress‐protective primed state, enabling rapid activation of protective pathways in response to stress without compromising performance under optimal conditions. This dual benefit underscores its potential as a tool for engineering climate‐resilient crops that combine productivity with stress tolerance (Colombatti et al., 2019; Wan & Li, 2006). Furthermore, our data reveal a reconfigured BR–ABA balance, in which Q12 represses BR signalling and the integrator OsDLT while selectively priming the stress‐protective arm of ABA signalling and attenuating its growth‐inhibitory arm. While BR repression is typically associated with growth arrest, OsQ12 plants exhibited remarkably enhanced biomass and length. This suggests that OsQ12 modulates downstream effectors, including CAM1 and ion transporters (OsHKT1 and OsSOS1), to optimise the metabolic cost of resistance, allowing the plant to maintain developmental momentum even in the presence of repressed growth‐promoting hormones. In this context, Q12 emerges as a master regulatory hub that integrates ROS signalling, antioxidant biosynthesis and ion transport pathways to orchestrate a rapid, energy‐efficient and multi‐layered defence against salinity (Figure 12). Our findings highlight the potential of engineering high‐level regulatory nodes to confer salt tolerance, a critical step toward developing climate‐resilient rice varieties and ensuring global food security (Sackey et al., 2025).

**Figure 12:**
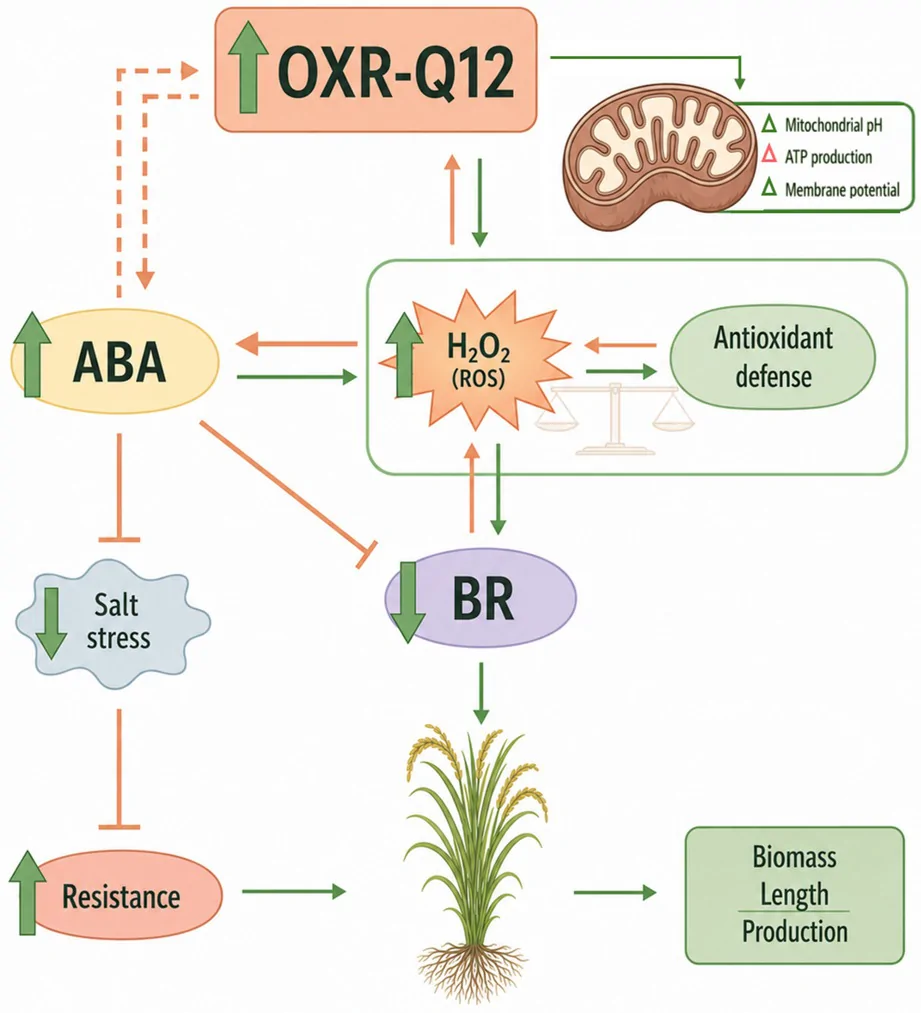
Proposed model: redox‐driven rewiring of ABA–BR crosstalk uncouples growth from salt defence in OsQ12 rice. The chimeric protein Q12, formed by the regulatory N‐terminus of AtOXR1 fused to the catalytic TLDc domain of AtOXR2, localises to mitochondria. There, lowered catalase (CAT) activity permits a controlled, non‐toxic rise in hydrogen peroxide (H_2_O_2_), together with higher superoxide dismutase (SOD) activity and an expanded, more reduced glutathione pool; these establish a primed basal redox state in which mitochondrial H_2_O_2_ acts as the main upstream signal. This redox state sits causally upstream of the downstream responses: scavenging H_2_O_2_ with exogenous ascorbate (ASC) reverts the OsQ12 phenotypes (seedling growth, salt tolerance and leaf erectness), shown here by the reversal arrow. Downstream, Q12 splits the hormonal output into two arms. In the growth arm, Q12 attenuates brassinosteroid biosynthesis and signalling (reduced *OsBRD1*, *OsBZR1*, *OsDLT*, *OsILI1*) and blunts ABA‐mediated inhibition of germination and seedling growth, giving erect leaves, a smaller flag leaf angle (FLA) and sustained biomass. In the protective arm, salinity drives strong induction of stress‐responsive genes (*OsNCED3/4*, *OsDREB2A*) alongside adjustment of ion transport (*OsHKT1*, *OsSOS1*), which confers salinity and drought tolerance. The combined output is an OsQ12 plant with erect architecture, more productive tillers, a larger root system with greater hydraulic capacity and higher biomass and grain yield than WT Kitaake, achieved without the growth cost normally imposed by the growth‐defence trade‐off. Solid arrows denote experimentally supported relationships; dashed arrows and boxes denote predicted or hypothetical steps.

We carried out the present characterisation under controlled growth chamber and greenhouse conditions, assessing salt tolerance at the seedling stage, when rice is most sensitive to salinity (Munns & Tester, 2008; Sackey et al., 2025). This study does not include multi‐season or multi‐site field trials, and the performance of OsQ12 rice under reproductive‐stage stress in the field remains untested. Such field evaluation requires trials across multiple seasons and locations, which were beyond the scope of this mechanistic study. Reproductive‐stage tolerance and the preservation of reproductive yield under both water deficit and salinity were established in the Arabidopsis system (Figure 3), in which Q12 lines retained seed yield above that of the WT under each stress. Stage‐resolved, multi‐season field evaluation across the seedling and reproductive windows, with direct quantification of yield loss or maintenance under salinity and drought, is the necessary next step to translate these findings into agronomic gain (Negrão et al., 2017; Roy et al., 2014).

Our *in silico* analyses place OXR2/Q12 alongside proton pump and energy/redox machinery: the V‐ATPase in both homologues, the TOR subunit LST8 and the PGR5 and NDH components. This recovers, in plants, the V‐ATPase‐TOR links that had been described only in yeast and mammals. We treat them as predictions to be validated rather than as established interactions, so we keep them in the text and in Table S3. The VHA‐C/DET3 candidate is worth highlighting on its own merits: it is a documented node that ties V‐ATPase activity to brassinosteroid signalling, guard‐cell ABA and Ca^2+^ responses and salinity tolerance (Allen et al., 2000; Schumacher et al., 1999), a plausible way for a proton pump and pH function to reach the hormonal outputs that define the Q12 phenotype. A limitation of this study is that the molecular partners of Q12/OXR2 are based on *in silico* functional association predictions rather than direct experimental evidence. Validating them will require dedicated biochemical work, which we have prioritised for future research.

As a working hypothesis for future research (Figure 12), we propose that mitochondrially targeted Q12 acts as an organellar rheostat that modulates the mitochondrial proton gradient (matrix pH and membrane potential, ΔΨ*m*) and ATP production. This establishes a primed basal redox state, characterised by elevated yet non‐toxic H_2_O_2_ accumulation and an expanded, highly reduced glutathione pool. This primed state would feed an ascorbate‐reversible retrograde H_2_O_2_ signalling cascade that reshapes the antagonistic crosstalk between the BR and ABA pathways. While stress‐induced ABA signalling normally overrides BR promotion in WT plants to arrest cell expansion and growth, Q12 expression selectively dampens this growth‐inhibitory brake. Consequently, BR‐dependent developmental signals prevail at the LJ and vegetative tissues, sustaining upright leaf posture and vegetative biomass even under challenging conditions. By functionally uncoupling stress tolerance from metabolic arrest through this proposed, redox‐mediated mechanism, Q12 may bypass the evolutionary growth–defence trade‐off, providing a molecular framework to overcome the physiological constraints that limit crop productivity (Figure 12).

## MATERIAL AND METHODS

### Plant material and growth conditions

#### Arabidopsis thaliana

*Arabidopsis thaliana* ecotype Columbia (Col‐0) plants were grown in 7‐cm pots containing a 2:2:1 (v/v/v) mixture of peat, perlite, and vermiculite, in growth chambers at 22–24°C under a long‐day (LD) photoperiod (16 h light/8 h dark) with a light intensity of 80–100 μmol m^−2^ s^−1^. Plants were watered regularly with 0.5X Hoagland solution (Hoagland & Arnon, 1950). For root biomass analysis, plants were grown in sand and sub‐irrigated with the same nutrient solution. For *in vitro* experiments, surface‐sterilised seeds were sown on Petri dishes containing 0.5X Murashige and Skoog (MS) medium supplemented with 0.8% (w/v) agar. Specific growth conditions for each assay are detailed in the corresponding sections.

#### Oryza sativa

Rice seedlings (*Oryza sativa* L. ssp. japonica cv. Kitaake) were transplanted into 8‐L or 20‐L pots containing GROW MIX MASTER E substrate. Plants were cultivated either in a growth chamber (28 ± 2°C, LD photoperiod, 140 μmol m^−2^ s^−1^) or in a greenhouse in Santa Fe, Argentina (31°38′17.1″ S, 60°40′01.8″ W) under natural light and ambient temperatures (12–35°C). From the fifth‐leaf stage (V5) onwards, plants were flooded with a solution of Hakhaphos® 18–18–18 (0.5 g/L) supplemented with iron, as described by Trionfini et al. (2023). For hydroponic culture, surface‐sterilised seeds were germinated under agitation in liquid 0.5X MS medium at 28°C under a 16 h light/8 h dark photoperiod. Seedlings were then transferred to a custom hydroponic system containing a nutrient solution of Hakhaphos® 18‐18‐18 (133 mg/L) and iron (0.5 g/L) (Figure S6).

### Generation of the chimeric protein and cloning into the binary vectors

To obtain Arabidopsis and rice plant lines that constitutively express the chimeric protein OXR‐Q12 (Q12) under the control of the 35S CaMV promoter, cDNA encoding AtOXR1 (At4g39870) and AtOXR2 (At2g05590) was obtained from Arabidopsis mature leaf total RNA. The N‐terminal portion of the chimeric protein, ranging from the start codon to the aa at position 202 of AtOXR1, and the C‐terminal part, containing the AtOXR2 portion from aa 128 to 303, were amplified by PCR using specific primers (see Table S1 for primer sequences and Figure S1 for protein sequences). Partial fragments were hybridised, and the entire artificial coding region of the chimeric protein was obtained by overlapping PCR. We cloned it into modified versions of the binary vectors pBi121 (Chen et al., 2003) and pCambia1302 (Curtis & Grossniklaus, 2003) to express Q12 in Arabidopsis and rice, respectively. To obtain plants expressing a fusion of Q12 to monomeric red fluorescent protein (mRFP) (35S: Q12‐mRFP), the Q12 coding region was first cloned into the pENTR3c vector (Thermofischer) and then transferred to the destination binary vector pGWB554 (Nakagawa et al., 2007).

### Generation of transgenic Arabidopsis and rice lines

*Agrobacterium tumefaciens* strain LBA4404 was transformed with the generated constructs, and transgenic Arabidopsis AtQ12 lines were generated by floral dip (Clough & Bent, 1998) to study the impact of Q12 expression and its subcellular localisation. Rice plants expressing the Q12 protein (OsQ12) were generated via *A*. *tumefaciens* EHA105‐mediated transformation, following the protocol established by Main et al. (2015), with modifications as described in Raineri et al. (2023). Ten positive independent Arabidopsis and rice lines were identified by resistance to kanamycin (0.050 mg/mL) or hygromycin (50 mg/L), respectively, and confirmed by PCR amplification of genomic DNA using specific primers (Table S2). Three selected Arabidopsis (AtQ12 L1, L2 and L3) and rice (OsQ12 L1, L3 and L13) lines were used to obtain homozygous plants for phenotypic analysis and molecular characterisation.

### Arabidopsis and rice phenotypic characterisation

Arabidopsis phenotypic characterisation was performed according to the parameters established by Boyes et al. (2001), using 15 individual plants per genotype per biological replicate. We measured the same parameters as those reported by Colombatti et al. (2019).

Rice seeds were surface‐sterilised according to Main et al. (2015), germinated in flasks containing 0.5X MS with gentle shaking, and then placed in an environmentally controlled growth chamber for 3 days. Three homozygous independent lines were used for further analyses. The homozygous transgenic lines were multiplied in a greenhouse. Phenotypic analysis throughout the life cycle was performed according to the parameters described by International Rice Research Institute (IRRI) (2002), using 10 plants per genotype and per biological replicate.

### Histological analysis of rice roots

Rice roots collected from hydroponically grown 30‐day‐old seedlings were fixed in 70% ethanol for 48 h. Freehand cross‐sections were made under a stereo microscope (Leica EZ4), deposited on slides (25.4 × 76.2 mm), and rinsed with 50% bleach, then washed five times with H_2_O. Samples were stained with a successive double astra‐safranin blue stain (Arend et al., 2008). Preparation, imaging and analysis were performed as described by Castro‐Estrada et al. (2025).

### Stress treatments

#### Arabidopsis thaliana

Water deficit assays were performed under controlled greenhouse conditions. Plants were grown as mentioned above. After 21 days of growth, irrigation was withheld until the soil water content reached approximately 40% of field capacity, as monitored gravimetrically (Claeys et al., 2014; Verslues et al., 2006). Control plants were watered to full field capacity throughout the experiment.

For salinity stress, plants were grown under the same environmental conditions described above. At 21 DAG, pots were irrigated with 100 mM NaCl for two consecutive days to allow acclimation, followed by irrigation with 150 mM NaCl for 10 days. Salinity was imposed by adding a salt solution to the soil until saturation, and the pots were subsequently maintained at field capacity at the same salt concentration. Control plants received water without NaCl (Munns, 2002; Munns & Tester, 2008). Damage markers, including ion leakage and ROS accumulation, were evaluated following established protocols (Blum & Ebercon, 1981; Jambunathan, 2010).

#### Oryza sativa

For *salinity stress treatments*, rice seeds were surface‐sterilised, germinated in flasks containing 0.5X MS medium with gentle shaking, and then placed in an environmentally controlled growth chamber, as mentioned above. Once the seeds had uniformly germinated, they were transferred to a rubber net stretched over an 8.5 L plastic pot containing 0.5X Hoagland solution (Control) and the same solution with 90 mM NaCl added. For phenotypic analysis, 6‐day‐old seedlings were photographed and evaluated using ImageJ. All assays were performed under the same photoperiod and humidity conditions and repeated three times. For marker gene expression related to salt stress, seedlings were grown for 8 days under control conditions and then transferred to a solution containing 200 mM NaCl for 6 h.

To evaluate *the effect of ABA on seed germination*, 50 seeds per replicate were used from each line. Seeds were imbibed in Petri dishes containing 10 mL of sterile distilled water and held at 4°C for 48 h. The water was replaced with ABA solutions (0, 1 or 5 μM) and seeds were transferred to a growth chamber under a 16 h light/8 h dark photoperiod at 28°C. Germination, scored as radicle emergence, was recorded every 24 h, and the germination rate was calculated as the number of germinated seeds over the total number of seeds analysed (adapted from Li et al., 2021). Shoot and primary root length were measured at 10 days after sowing (DAS) using ImageJ.

To assess *ABA sensitivity during post‐germination growth*, 24 h‐germinated seeds were transferred to glass containers with 0.5X liquid MS medium supplemented with 0 or 1 μM ABA and grown under a 16 h light/8 h dark photoperiod at 28°C for 10 days. Shoot length, root length and LJ angle were measured using ImageJ. The LJ angle response to ABA was expressed as the ratio of the angle under ABA to that under control conditions.

### Ascorbate treatments

To probe the contribution of the elevated basal H_2_O_2_ pool to the OsQ12 phenotype, seedlings were grown in the presence of ascorbate (ascorbic acid, ASC). Seedlings were first grown for 4 days in liquid 0.5X MS medium or in 0.5X MS containing 120 mM NaCl, under the conditions described above. The medium (0.5X MS or 0.5X MS + 120 mM NaCl) was then renewed daily for six consecutive days, each time adding 5 mM ASC. We performed renewals at the onset of the dark period to limit ascorbate photodegradation. Shoot and root lengths were recorded on Day 10 after sowing. For the LJ assay, 1‐cm segments isolated from 10‐day‐old plants, as described above, were incubated for 48 h with 50 mM ascorbate before the LJ angle was measured with ImageJ. We performed all assays with 30 seedlings per genotype and treatment in each of three independent biological replicates.

### Lamina joint (LJ) inclination assay and hormone treatments

We performed the LJ assay as described by Trionfini et al. (2023), with minor modifications. Segments consisting of 1 cm of the second leaf blade, the LJ and 1 cm of the leaf sheath were excised from seedlings, floated on 0.5X MS liquid medium in a Petri dish with the addition of 200 nM BL and/or 100 μM ABA, and placed in a growth chamber for 24 h to evaluate the LJ under normal conditions and for 48 h for BL and ABA evaluations. For the ascorbate treatment, excised LJ segments were floated on 0.5X liquid MS medium without (Control, ‐ASC) or with 50 mM ascorbate (+ASC) for 48 h. LJ images were taken with a light microscope (Leica MZ10F), and the LJ angle was measured in ImageJ. Twenty‐five seedlings were used for each condition, and all experiments were repeated three times.

### RNA isolation and RT‐qPCR analysis

Total RNA (1 μg) was extracted from Arabidopsis or rice leaves as described by Welchen et al. (2012). RNA quality and quantity were evaluated using a NanoDrop 2000 (Thermo Scientific, Waltham, MA, USA) system. The RT‐qPCR analysis was performed as described by Castro‐Estrada et al. (2025). Gene expression was calculated by the 2^−ΔΔCt^ method (Pfaffl, 2001), using *ACTIN2/8* as the reference gene for Arabidopsis AtQ12 and *UBIQUITIN 10* (*OsUBQ10*) for rice (Almas & Kamrodi, 2018). Three to five biological replicates were used in each experiment, and the bars represent the mean + SD. All primers used for RT‐qPCR analyses are listed in Table S2.

### Measurement of photosynthetic parameters

Leaf photosynthetic parameters were measured using a LiCOR 6400‐XT portable photosynthetic system (LI‐COR Inc.). The analysis was conducted in accordance with Colombatti et al. (2019). Before measurements, each leaf was adapted to darkness for 10 min, and we took 10 measurements for each genotype using different plants. Chlorophyll fluorescence parameters were calculated according to Maxwell and Johnson (2000) and Baker (2008).

### Membrane permeability measurements (ion leakage)

Membrane permeability was measured by electrolyte leakage. Leaf discs were placed in a beaker containing 10 mL of distilled water, and the conductivity of the solution was measured after 20 h at 25°C using a HI 2004 (Hanna Instruments, Padova, Italy) conductivity meter. After measuring the initial electrical conductivity of the solution (C1), the samples were autoclaved at 121°C for 20 min to induce complete electrolyte leakage, and the final conductivity (C2) was measured at room temperature. The relative ion leakage was calculated as a percentage using the formula: Ion Leakage (%) = (C1/C2) × 100.

### ROS detection by histochemical staining

For ROS detection, the protocol described by Thordal‐Christensen et al. (1997) was adapted. OsQ12 and control (Kitaake) plant tissues were incubated in a 1 mg mL^−1^ 3,3′‐Diaminobenzidine (DAB) solution for3 h in the dark at room temperature, then clarified with 70% ethanol and immediately photographed. H_2_O_2_ content was detected as a dark‐brown precipitate. The *in situ* accumulation of superoxide radicals (O_2_^•−^) in leaf tissue was visualised using nitroblue tetrazolium (NBT) staining. Rice seedlings were vacuum‐infiltrated for 15 min with a 0.1% (w/v) NBT solution in 10 mM potassium phosphate buffer (pH 7.8) and incubated in the dark at room temperature for 6 h. Following incubation, the staining reaction was stopped, and chlorophyll was removed by immersing the tissues in 80% (v/v) ethanol and incubating at 70°C for 2 h, with several ethanol changes until the tissues were fully decolourised. The formation of a dark blue formazan precipitate, resulting from the reaction of NBT with superoxide radicals, was documented by analysing the destained roots.

### Determination of antioxidant capacity by enzymatic activity quantitation

#### Superoxide dismutase (SOD) activity assay

*In‐gel SOD activity assay*: total soluble proteins were extracted from the rosette leaves of 30‐day‐old plants. Approximately 100 mg of leaf tissue was ground to a fine powder in liquid nitrogen and homogenised in 500 μL of extraction buffer. The homogenate was centrifuged at 13000× ***g*** for 20 min at 4°C, and the supernatant was collected for analysis. Protein concentration was determined using the Bradford method. For the SOD activity assay (zymogram), equal amounts of protein (30 μg) were separated by native polyacrylamide gel electrophoresis (PAGE) on a 12% gel. After electrophoresis, the gel was incubated in the dark for 30 min in a staining solution containing 50 mM potassium phosphate buffer (pH 7.8), 2.5 mM NBT and 1 mM EDTA. The gel was then briefly washed, immersed in a developing solution, and illuminated on a light box until achromatic bands representing SOD activity appeared against a purple formazan background. For a loading control, a parallel gel was run and stained with Coomassie Brilliant Blue R‐250.

*Soluble enzymatic SOD activity* was determined using the method of Beauchamp and Fridovich (1971) with modifications. Extracts prepared in phosphate buffer (50 mM, pH 7.0) were incubated in a reaction mixture containing NBT, riboflavin and methionine. Superoxide radicals were generated under illumination, and the reduction of NBT was followed at 560 nm. SOD activity was calculated from its ability to inhibit NBT reduction, with one unit defined as the amount of enzyme required to achieve 50% inhibition under the assay conditions. Results were normalised to protein content and expressed as units per mg protein.

### Catalase (CAT)

CAT activity was assayed as described by Aebi (1984). Fresh tissue was homogenised in cold phosphate buffer (50 mM, pH 7.0) and centrifuged at 12000 ***g*** for 15 min at 4°C. The reaction mixture contained hydrogen peroxide (H_2_O_2_) as substrate, and the decomposition of H_2_O_2_ was monitored spectrophotometrically by the decrease in absorbance at 240 nm. Enzyme activity was expressed as units per mg protein, where one unit corresponds to the amount of enzyme decomposing 1 μmol of H_2_O_2_ per minute under assay conditions.

### Glutathione content

Glutathione content was determined in tissue extracts using a recycling assay with 5,5′‐dithiobis‐(2‐nitrobenzoic acid) (DTNB) and glutathione reductase, following the formation of the yellow product (TNB) at 412 nm in a microplate reader; briefly, approximately 100 mg of fresh tissue was homogenised in cold extraction buffer (100 mM sodium phosphate, pH 7.5, 1 mM EDTA), centrifuged at 12000 ***g*** for 10 min at 4°C, and the supernatant used for analysis, with total glutathione measured in the presence of NADPH (0.2 mM) and glutathione reductase (1 U/mL), oxidised glutathione (GSSG) quantified after preincubation of samples with 2‐vinylpyridine (10 mM, 1 h, room temperature) to derivatise reduced glutathione, and reduced glutathione (GSH) calculated by subtracting GSSG from total glutathione, with results expressed as ng per mg protein, and each determination performed in technical triplicate (Queval & Noctor, 2007).

### Total phenolic content (TPC) and total flavonoid content (TFC)

TPC was determined using the Folin–Ciocalteu colourimetric method adapted for microplate analysis, as described by Sumczynski et al. (2015). Briefly, aliquots of the sample extract were mixed with Folin–Ciocalteu reagent and sodium carbonate solution, incubated at room temperature to allow colour development, and the absorbance was measured at 765 nm using a plate reader. Gallic acid was used to generate the calibration curve, and results were expressed as mg gallic acid equivalents (GAE) per kg of sample dry weight.

TFC was quantified using the aluminium chloride colorimetric assay described by Sumczynski et al. (2015). In short, sample extracts were combined with aluminium chloride reagent and potassium acetate, incubated at room temperature, and absorbance was recorded at 415 nm. Rutin was used as the standard for calibration, and values were expressed as mg rutin equivalents (RUT) per kg of sample dry weight.

### Chlorophylls and anthocyanins

Chlorophylls were extracted in 80% acetone according to the protocol described by Lichtenthaler (1987). Pigment concentrations were determined spectrophotometrically, and chlorophyll a, chlorophyll b, and total chlorophyll contents were calculated using the extinction coefficients and equations provided in Methods in Enzymology (Lichtenthaler, 1987).

Anthocyanins were extracted with an acidified methanolic solution consisting of 6 M HCl, water, and methanol (7:23:70 (v/v/v)). Before extraction, leaf tissue was immersed in liquid nitrogen for 5 min to facilitate cuticle perforation and membrane disruption. Samples were incubated in the extraction solution in the dark at 4°C for 24 h. Anthocyanin concentration was subsequently quantified spectrophotometrically as A_530_–0.24 × A_653_, following the method described by Murray and Hackett (1991).

### Proline content

Free proline content was determined using the acid–ninhydrin method (Hayat et al., 2012). Approximately 100 mg of fresh leaf tissue from 25‐day‐old plants was homogenised in 1.5 mL of 3% (w/v) aqueous sulfosalicylic acid. The homogenate was centrifuged at 10000× ***g*** for 10 min. A 100‐μL aliquot of the supernatant was mixed with 100 μL of glacial acetic acid and 100 μL of acid–ninhydrin reagent (1.25 g ninhydrin dissolved in 30 mL glacial acetic acid and 20 mL of 6 M phosphoric acid). The mixture was incubated at 100°C for 1 h. The reaction was terminated by placing the tubes in an ice bath. The reaction chromophore was extracted with 500 μL of toluene by vigorous vortexing. After phase separation, we measured the absorbance of the upper toluene phase at 520 nm using a spectrophotometer, with toluene as the blank. Proline concentration was calculated from a standard curve prepared with known concentrations of L‐proline and expressed as μmol per gram of FW.

### Confocal laser scanning microscopy

For confocal laser scanning microscopy (CLSM), roots of 15‐day‐old plants constitutively expressing the Q12‐mRFP protein in the mt‐gk background (Nelson et al., 2007) were imaged with a Leica TCS SP8 or a Zeiss LSM880 microscope with excitation at 488 nm and detection at 498–531 nm for GFP or excitation at 561 nm and detection at 587–620 nm for mRFP. Chlorophyll autofluorescence was excited at 633 nm and detected at 650–720 nm. For co‐localisation analysis, intensity‐scatter plots were generated using the Coloc 2 and Colocalization Threshold plugins in Fiji (Schindelin et al., 2012).

### Analysis of the structure of the Q12 chimeric protein

The structure of the Q12 chimeric protein was analysed using AlphaFold3 (https://alphafoldserver.com/welcome; Abramson et al., 2024) to predict its structure from the aa sequences of Arabidopsis OXR family members (Colombatti et al., 2019). Comparisons of the structures were conducted using Chimera 8 (https://www.cgl.ucsf.edu/chimera/; Pettersen et al., 2004) and ProtParam (https://web.expasy.org/protparam/; Gasteiger et al., 2005). The C‐terminal TLDc domains were defined as residues 128–303 in AtOXR2 and 203–378 in AtQ12; the structures were aligned and superposed using MatchMaker in UCSF Chimera, and the RMSD was calculated over the Cα atoms of these residue ranges. The reported RMSD is 0.386 Å, based on 165 aligned Cα pairs. MatchMaker was executed with the default parameters (command used: matchmaker #0 #1). The same analysis was extended to the closest native rice orthologue OsOXR2 (Os07g0438700; B9FWZ0) using a global Needleman–Wunsch algorithm with the BLOSUM62 substitution matrix (gap‐opening penalty: 10, gap extension: 0.5; Biopython 1.8). Alignments were computed for both the full‐length proteins and, separately, the TLDc domains (OXR‐Q12 residues 203–378, derived from AtOXR2; OsOXR2 residues 165–329). Per cent identity and per cent similarity were calculated, and physicochemical parameters were derived using the ProtParam module of Biopython (https://biopython.org/wiki/ProtParam).

### *In silico* prediction of putative OXR2 functional associates

To explore the molecular context in which OXR2 proteins may operate, genome‐wide functional association networks were retrieved for the rice protein OsOXR2 (UniProt B9FWZ0; locus Os07g0438700) and its Arabidopsis homologue AtOXR2 (UniProt Q9AT60; locus AT2G05590). FunCoup 5 (https://funcoup.org/; Persson et al., 2021) builds these networks by combining several types of evidence, including co‐expression, physical interactions, shared transcription factor binding, phylogenetic profiles, and subcellular co‐localisation and assigns each pair a probabilistic functional coupling (PFC) score. STRING v12 (https://string‐db.org/; Szklarczyk et al., 2023) compiles known and predicted associations from curated databases, high‐throughput experiments, co‐expression, genomic context and text mining, reporting a combined confidence score between 0 and 1. Interolog‐based predictions, including modelled 3D complex structures, were also retrieved from PlaPPISite (http://zzdlab.com/plappisite/; Yang et al., 2020). Because functional coupling encompasses associations broader than direct physical binding, all retrieved partners are reported as predicted functional associates rather than validated interactors. As a reference for experimental evidence, the IntAct (https://www.ebi.ac.uk/intact/home; Orchard et al., 2014) and BioGRID (https://thebiogrid.org/; Oughtred et al., 2021) molecular interaction repositories were also queried for both proteins.

### Statistical analysis

Data were analysed using one‐way ANOVA, followed by Dunnett's multiple comparisons test, to compare each treatment group with the control. All pairwise comparisons shown in the figures were analysed using Dunnett's test. For experiments with a factorial design (genotype combined with a treatment, namely, control versus salinity stress, ±ABA, or ± ascorbate), data were analysed by one‐way ANOVA, comparing treatments within each genotype. A significant effect was taken as evidence of a treatment‐dependent response. Statistical analyses were performed with InfoStat v.2013 for Windows (http://www.infostat.com.ar). Data are expressed as mean ± SD, with significance indicated by an asterisk (*) for *P* < 0.05. The number of biological replicates for each experiment is provided in the corresponding figure legends.

## AUTHOR CONTRIBUTIONS

BAE performed the experiments in rice and prepared the Figures. TP generated the OsQ12 rice lines and conducted their initial characterisation. CG generated and characterised the Arabidopsis AtQ12 lines. CM generated the OsQ12 rice plants. BAE and WE conceived and designed the experiments. WE led the work, wrote the manuscript, and contributed to the design of the figure.

## CONFLICT OF INTEREST

The authors declare no conflicts of interest.

## Supporting information


**Table S1.** List of oligonucleotides used for cloning in this work.
**Table S2.** List of oligonucleotides used for qRT‐PCR in this work.
**Table S3.** Predicted interactor partners for AtOXR2 and OsOXR2.

**Figure S1.** Sequence of OXR family proteins AtOXR1, AtOXR2 and the chimeric protein OXR‐Q12 (Q12). (A) Amino acid sequence of AtOXR1 (At4g39870, Q8GUN9), a full‐length OXR family protein containing an N‐terminal regulatory region (coral) and a C‐terminal TLDc domain (terracotta). (B) Amino acid sequence of AtOXR2 (At2g05590, Q9AT60), a full‐length OXR family protein containing an N‐terminal regulatory region (light violet) and a C‐terminal TLDc domain (dark violet). (C) Sequence of the chimeric protein OXR‐Q12, generated by fusing the N‐terminal region of AtOXR1 with the TLDc domain of AtOXR2. Colour coding indicates the origin and identity of each region. Accordingly, OXR‐Q12 displays the coral AtOXR1‐derived N‐terminus fused to the dark violet AtOXR2‐derived TLDc domain plus the C‐terminal residues lying outside the TLDc domain (grey).

**Figure S2.** Pairwise sequence alignment of the engineered chimera OXR‑Q12 and its TLDc‑donor parent AtOXR2. Global pairwise alignment of the full‑length OXR‑Q12 (Q12) and AtOXR2 amino‑acid sequences. Residues are coloured by region: the Q12 N‑terminal region derived from AtOXR1 (coral), the native AtOXR2 N‑terminal region (light violet), the shared TLDc domain, which is identical in Q12 and AtOXR2 (dark violet), and the C‑terminal residues lying outside the TLDc domain (grey). The thin bars above the Q12 row and below the AtOXR2 row indicate the same regions. The consensus line marks identical residues (asterisk) and conservative substitutions. The chimera Q12 and AtOXR2 share 68.2% identity and 77.5% similarity over the full alignment. The AtOXR2‑derived C‑terminal half of Q12 (residues 203–378) is 100% identical to AtOXR2 (residues 128–303), confirming that the complete TLDc‑containing module was transferred intact during chimera construction. The violet asterisk indicates the four Cysteines exposed on the protein surface, likely accessible for redox reactions, as reported by Colombatti et al. (2019). The two proteins diverge in their N‑terminal regions, and the gap in the AtOXR2 sequence corresponds to the additional AtOXR1‑derived N‑terminus carried by Q12. Residue positions are indicated at the end of each row.

**Figure S3.** Molecular characterisation of the AtQ12 Arabidopsis lines used in this study. Q12 transcript levels in AtQ12 Arabidopsis lines are shown. Expression levels are normalised to those of the AtQ12 line, which has the lowest expression and was set arbitrarily to ‘1’ (#5). Arrows indicate the transgenic lines used in this work.

**Figure S4.** *In silico* structural and physicochemical characterisation of the chimeric protein Q12 and OsOXR2. (A) Schematic representation of the domain architecture of engineered chimera OXR‐Q12 (Q12) and OsOXR2. The N‐terminal domain of AtOXR1 (coral) was fused to the C‐terminal TLDc domain‐containing region of AtOXR2 (dark violet). The native N‐terminus of OsOXR2 is shown in light teal, and the C‐terminus of OsOXR2, which also contains a TLDc domain, is represented in deep teal. The accompanying table provides Gene Identifier (GI) codes, protein sizes in amino acids (aa), UniProt accession numbers, and instability indices (II) for the Proteins. (B–C) Predicted tertiary structures of full‐length Q12 (B) and OsOXR2 (C), generated using the AlphaFold 3 algorithm (Abramson et al., 2024). Mean pLDDT values: AtQ12 = 65.6 and OsOXR2 = 63.1. The models illustrate the structurally distinct N‐terminal regions and the conserved fold of the C‐terminal TLDc domain. (D) Structural superposition from Q12 and OsOXR2. Alignment and visualisation were performed with UCSF Chimera (Pettersen et al., 2004); RMSD = 0.477 Å. The surrounding N‐terminal domain and unstructured regions are shown as thin traces for structural context. (E) Pairwise BLOSUM62 alignment between Q12 and OsOXR2 revealed 48.9% amino acid identity across the full‐length proteins, increasing to 69.7% identity and 80.6% similarity within the conserved TLDc domain. Asterisks correspond to three of the four surface‐exposed cysteine residues, proposed to participate in redox reactions (Colombatti et al., 2019), that are conserved within the OsOXR2 domain.

**Figure S5.** Molecular characterisation of the OsQ12 rice lines used in this study. Q12 transcript levels in OsQ12 lines are shown. Expression levels are normalised to those of the OsQ12 line, which has the lowest expression and was set arbitrarily to ‘1’ (#7). Arrows indicate the transgenic lines used in this work.

**Figure S6.** Experimental growth system for rice plant cultivation. The diagram illustrates the cultivation pipeline from germination to maturity, divided into stages within a controlled growth chamber and a greenhouse. (A) Germination and early seedling growth (0–10 DAG) in glass flasks containing liquid medium, placed on an orbital shaker for gentle agitation within a controlled growth chamber. (B) Vegetative growth stage (10–30 DAG), where seedlings are transferred to a hydroponic system within the growth chamber. (C) An alternative or subsequent vegetative growth stage (15–30 DAG) in a larger‐scale hydroponic system, with plants supported by foam holders, located in the greenhouse. (D) Reproductive and maturation stage (30–180 DAG). Plants are transferred to soil in individual pots and grown to maturity in the greenhouse to assess panicle development, seed set and final agronomic traits.

**Figure S7.** Absolute germination and seedling growth of OsQ12 rice lines exposed to ABA treatment. (A) Germination rate (%) at 0, 1 and 5 μM ABA was scored 3 days after sowing (DAS). (B) Germination rate at 3 DAS, on 5 μM ABA relative to control. (C) Shoot, (D) root and (E) total seedling length, at 0 and 1 μM ABA, scored 10 DAS, measured in WT (Kitaake), L1, L3 and L13 OsQ12 lines. Graphs show the mean ± SD of three independent biological replicates. Asterisks indicate *P* < 0.05 (one‐way ANOVA, Dunnett's test against Kitaake).

**Figure S8.** Analysis of the impact of ascorbate on salinity stress tolerance. (A) Aerial and (B) root length under salinity (120 mM NaCl), expressed as the NaCl/MS ratio, showing the salt tolerance of the OsQ12 lines. (C) Aerial and (D) root length under salinity in the presence of ascorbate (ASC 5 mM), expressed as the (NaCl+ASC)/MS ratio. Data are shown as: mean ± SD, *n* = 30. Asterisks (*) indicate a significant difference from Kitaake at *P* < 0.05 (one‐way ANOVA followed by Dunnett's *post‐hoc* test).

**Video S1.** Three‐dimensional rotational view of the AtOXR2 and Q12 structural models.

**Dataset S1.** Complete 3D structural datasets for AtOXR1, AtOXR2, and Q12 (including .pdb, .json files, pLDDT metrics, and the animation video).

## Data Availability

All relevant data can be found within the published article and its supporting material.
