# Supplementary material for: Expression of a chimeric OXR protein remodels rice architecture for enhanced grain yield by modulating redox and hormonal pathways
Source: Plant J. 2026 Nov 9;128(3):e71213. doi: 10.1111/tpj.71213 (PMC13748695; doi:10.1111/tpj.71213)
Supplement: Supplementary file 2 [file TPJ-128-0-s003.docx]

**Supplementary Material:**

**Supplementary Figure S1: Sequence of OXR family proteins AtOXR1, AtOXR2 and the chimeric protein OXR-Q12 (Q12). (A)** Amino acid sequence of AtOXR1 (At4g39870, Q8GUN9), a full-length OXR family protein containing an N-terminal regulatory region (coral) and a C-terminal TLDc domain (terracotta). **(B)** Amino acid sequence of AtOXR2 (At2g05590, Q9AT60), a full-length OXR family protein containing an N-terminal regulatory region (light violet) and a C-terminal TLDc domain (dark violet). **(C)** Sequence of the chimeric protein OXR-Q12, generated by fusing the N-terminal region of AtOXR1 with the TLDc domain of AtOXR2. Colour coding indicates the origin and identity of each region. Accordingly, OXR-Q12 displays the coral AtOXR1-derived N terminus fused to the dark violet AtOXR2-derived TLDc domain plus the C-terminal residues lying outside the TLDc domain (grey).

**Supplementary Figure S2: Pairwise sequence alignment of the engineered chimera OXR‑Q12 and its TLDc‑donor parent AtOXR2.** Global pairwise alignment of the full‑length OXR‑Q12 (Q12) and AtOXR2 amino‑acid sequences. Residues are coloured by region: the Q12 N‑terminal region derived from AtOXR1 (coral), the native AtOXR2 N‑terminal region (light violet), the shared TLDc domain, which is identical in Q12 and AtOXR2 (dark violet), and the C‑terminal residues lying outside the TLDc domain (grey). The thin bars above the Q12 row and below the AtOXR2 row indicate the same regions. The consensus line marks identical residues (asterisk) and conservative substitutions. The chimera Q12 and AtOXR2 share 68.2% identity and 77.5% similarity over the full alignment. The AtOXR2‑derived C‑terminal half of Q12 (residues 203-378) is 100% identical to AtOXR2 (residues 128-303), confirming that the complete TLDc‑containing module was transferred intact during chimera construction. The violet asterisk indicates the four Cysteines exposed on the protein surface, likely accessible for redox reactions, as reported by Colombatti *et al*. (2019). The two proteins diverge in their N‑terminal regions, and the gap in the AtOXR2 sequence corresponds to the additional AtOXR1‑derived N‑terminus carried by Q12. Residue positions are indicated at the end of each row.

**Supplementary Figure S3: Molecular characterisation of the AtQ12 Arabidopsis lines used in this study.** Q12 transcript levels in AtQ12 Arabidopsis lines are shown. Expression levels are normalised to those of the AtQ12 line, which has the lowest expression and was set arbitrarily to “1” (#5). Arrows indicate the transgenic lines used in this work.

**Supplementary Figure S4: In *silico* structural and physicochemical characterisation of the chimeric protein Q12 and OsOXR2**. **(A)** Schematic representation of the domain architecture of engineered chimera OXR-Q12 (Q12) and OsOXR2. The N-terminal domain of AtOXR1 (coral) was fused to the C-terminal TLDc domain-containing region of AtOXR2 (dark violet). The native N-terminus of OsOXR2 is shown in light teal, and the C-terminus of OsOXR2, which also contains a TLDc domain, is represented in deep teal. The accompanying table provides Gene Identifier (GI) codes, protein sizes in amino acids (aa), UniProt accession numbers, and instability indices (II) for the Proteins. **(B-C)** Predicted tertiary structures of full-length Q12 **(B)** and OsOXR2 **(C)**, generated using the AlphaFold 3 algorithm (Abramson *et al*., 2024). Mean pLDDT values: AtQ12 = 65.6 and OsOXR2 = 63.1. The models illustrate the structurally distinct N-terminal regions and the conserved fold of the C-terminal TLDc domain. **(D)** Structural superposition from Q12 and OsOXR2. Alignment and visualisation were performed with UCSF Chimera (Pettersen *et al*., 2004); RMSD = 0.477 Å. The surrounding N-terminal domain and unstructured regions are shown as thin traces for structural context. **(E)** Pairwise BLOSUM62 alignment between Q12 and OsOXR2 revealed 48.9% amino acid identity across the full-length proteins, increasing to 69.7% identity and 80.6% similarity within the conserved TLDc domain. Asterisks correspond to three of the four surface-exposed cysteine residues, proposed to participate in redox reactions (Colombatti *et al*., 2019), that are conserved within the OsOXR2 domain.

**Supplementary Figure S5: Molecular characterisation of the OsQ12 rice lines used in this study.** Q12 transcript levels in OsQ12 lines are shown. Expression levels are normalised to those of the OsQ12 line, which has the lowest expression and was set arbitrarily to “1” (#7). Arrows indicate the transgenic lines used in this work.

**Supplementary Figure S6: Experimental growth system for rice plant cultivation.** The diagram illustrates the cultivation pipeline from germination to maturity, divided into stages within a controlled growth chamber and a greenhouse. **(A)** Germination and early seedling growth (0-10 DAG) in glass flasks containing liquid medium, placed on an orbital shaker for gentle agitation within a controlled growth chamber. **(B)** Vegetative growth stage (10-30 DAG), where seedlings are transferred to a hydroponic system within the growth chamber. **(C)** An alternative or subsequent vegetative growth stage (15-30 DAG) in a larger-scale hydroponic system, with plants supported by foam holders, located in the greenhouse. **(D)** Reproductive and maturation stage (30-180 DAG). Plants are transferred to soil in individual pots and grown to maturity in the greenhouse to assess panicle development, seed set, and final agronomic traits.

**Supplementary Figure S7: Absolute germination and seedling growth of OsQ12 rice lines exposed to ABA treatment. (A)** Germination rate (%) at 0, 1 and 5 µM ABA was scored 3 days after sowing (DAS). **(B)** Germination rate at 3 DAS, on 5 µM ABA relative to control. (**C)** Shoot, **(D)** root and **(E)** total seedling length, at 0 and 1 µM ABA, scored 10 days after sowing (DAS), measured in WT (Kitaake), L1, L3 and L13 OsQ12 lines. Graphs show the mean ± SD of three independent biological replicates. Asterisks indicate *p* < 0.05 (one-way ANOVA, Dunnett’s test against Kitaake).

**Supplementary Figure S**8. **Analysis of the impact of Ascorbate on salinity-stress tolerance. (A)** Aerial and **(B)** root length under salinity (120 mM NaCl), expressed as the NaCl/MS ratio, showing the salt tolerance of the OsQ12 lines. **(C)** Aerial and **(D)** root length under salinity in the presence of ascorbate (ASC 5mM), expressed as the (NaCl+ASC)/MS ratio. Data are shown as: mean ± SD, n =30. Asterisks (*) indicate a significant difference from Kitaake at *p* < 0.05 (one-way ANOVA followed by Dunnett’s post-hoc test).

**Supplementary Video S1: Three-dimensional rotational view of the AtOXR2 and Q12 structural models.**

**Supplementary Table S1: List of oligonucleotides used for cloning in this work.**

**Supplementary Table S2: List of oligonucleotides used for qRT-PCR in this work.**

**Supplementary Table S3: Predicted interactor partners of AtOXR2 and OsOXR2.**

**Figure S1**

**Figure S2**

**Figure S3**

**Figure S4**

**Figure S5**

**Figure S6**

**Figure S7**

**Figure S8**

**Supplementary Table S1**. List of oligonucleotides used for cloning

| **Primer** | **Sequence** |
| --- | --- |
| *At1g-QFw* | GGC**AGATCT**GAAATGGGGAAACACAAATC |
| *At2g-QRv* | GGCGTCGACTTACGAGAGGTATTGAGAC |
| *Q12-QFw* | CAAGCAATTATACCTTTAACAGAGAGCTCTGTG |
| *Q12-QRv* | CAGAGCTCTCTGTTAAAGGTATAATTGCTTGCACTAC |

**Supplementary Table S2:** Oligonucleotides used for qRT-PCR in this work.

| **Primer** | **Sequence** |
| --- | --- |
| *AtqrtOXR2F* | GGCAACAAGTGGACCTTCTGAAAC |
| *AtqrtOXR2R* | GAGACGCATGTGCAAATCCCCAG |
| *OsABA2 Fw* | AGCATGCTGTGGTGGGTCTAAC |
| *OsABA2 Rv* | TTCACGCGTATCCCATGCTTCC |
| *OsAP2-39 FW* | ATCTGAGCCTCGGGATGT |
| *OsAP2-39 RV* | CACCACGGATGACGACGA |
| *OsBRD1 Fw* | TTCAATCCATGGAGGTGGCTGGAG |
| *OsBRD1 Rv* | TTCTACGGTGCCTACTTCCTTCCC |
| *OsbZIP23qFv* | GTCCAACTTGTCGGCTCATTCTC |
| *OsbZIP23qFw* | AGCAGGATGAAATGTTGGAGCAG |
| *OsBZR1-Fw* | GACCTCGTGGATCCTCTCGC |
| *OsBZR1-Rv* | CCATGGAGATAGAAGGGACGG |
| *OsCAM1-qrtFw* | TCGACAAGAACGGCGATGGATG |
| *OsCAM1-qrtRv* | CAAGGCCAAGAGAACGAGTCAC |
| *OsDLT Fw* | AGGGCCCATTTGTTCTTGATCC |
| *OsDLT Rv* | AGAATGGGTGGCTTCGCCTTAG |
| *OsDREB2A Fw* | ATGCTGTTTCGATTTGTGTCTTGC |
| *OsDREB2A Rv* | TCGCGTTCTCTTCTTCCTGACC |
| *OsGSK2-Fw* | TCCAGGGGTCTGTCATAGGG |
| *OsGSK2-Rv* | CCGTCATACCAGCTGATCCC |
| *OsHKT1.1-qRTFw* | CCTAAGTTGCCAACGGCGTTTC |
| *OsHKT1.1-qRTRv* | TCGACAGCACCGAACAATGTGAC |
| *OsILI1-Fw* | GTCTCGCTGAGGTTGTCCAC |
| *OsILI1-Rv* | AGTCTCAGGCTCGCAATGG |
| *OsMAPK5-Fw* | AGCAGCAGCGATCACAAATTGTGC |
| *OsMAPK5-Rv* | TATTAAAAAGGGAAGGGGGCGGCG |
| *OsNCED3 Fw* | TCAGCTGAGTCCACACTAGTCC |
| *OsNCED3 Rv* | TGGGCCCTCTCTCTCTCTATCTTG |
| *OsNCED4qFw* | GCACGGCACCTTCATTGG |
| *OsNCED4qRv* | CTGTTAATTCTTTTTACTGTTGCGTTCT |
| *OsPEX11-Fw* | GCGTCTACTACTTCCTCG |
| *OsPEX11-Rv* | GACTCCAGTTTGCCGATC |
| *OsPP108F* | CGCAGCTCCGACAACATCT |
| *OsPP108R* | GCATCAGCTGGGTGACACTCT |
| *OsPP2C49qFw* | GGCTTATTCTCTTCCTCCTCTAT |
| *OsPP2C49qRv* | AGTAAATTCTTTGCGACGATGAT |
| *OsPYL/RCARqFW* | AGGCTCCTGTTTACCTCGTGTG |
| *OsPYL/RCARqRv* | TGACGAACGGCTTGTACCTCTG |
| *OsRab16AqFw* | GAGTGTGAATGTGTGATGGCGTTC |
| *OsRab16AqRv* | CAGTACAAACAACACGCAGACACG |
| *OsSERF1-Fw* | GAGTGAGGAGCTCATTGTTTACGA |
| *OsSERF1-Rv* | ACATCAAAATTTCCATGTCATCTA |
| *OsSOS1-Fw* | ATACTGAGTGGGGTTGTTATTGC |
| *OsSOS1-Rv* | AAAGGTAAATTTCAAAAGGTACATGG |
| *OsUBQ 10-Fw* | TGGTCAGTAATCAGCCAGTTTGG |
| *OsUBQ 10-Rv* | GCACCACAAATACTTGACGAACAG |
| *OsWRKY24 -FW* | CACTTTGACTGGAGGCCGGA |
| *OsWRKY24 -Rv* | GCGCCGTCTGAAACGAGAAG |

**Supplementary Table S3: Predicted interactors partners for AtOXR2 and OsOXR2.**

| **Query protein (UniProt / locus)** | **Species** | **Partner (symbol)** | **Predicted partner — description** | **Partner UniProt** | **Partner locus** | **Database** | **Confidence score** | **Notes** | **Interaction Network** |  |
| --- | --- | --- | --- | --- | --- | --- | --- | --- | --- | --- |
| OsOXR2 (B9FWZ0 /  Os07g0438700) | *O. sativa* | Q65XE2 | OsTON2/TONNEAU2 (EF-hand domain-  containing protein) | Q65XE2 | Os05g0149800 | FunCoup5 | 0.893 | High-confidence functional  coupling (PFC) |  | |
| OsOXR2 (B9FWZ0 /  Os07g0438700) | *O. sativa* | Q5VPE9 | Leucine carboxyl methyltransferase 1  homolog | Q5VPE9 | Os06g0140100 | FunCoup5 | 0.890 | High-confidence functional  coupling (PFC) |  |  |
| OsOXR2 (B9FWZ0 /  Os07g0438700) | *O. sativa* | Q0DJF0 | Nucleolar factor 1 | Q0DJF0 | Os05g0295100 | FunCoup5 | 0.890 | High-confidence functional  coupling (PFC) |  |  |
| OsOXR2 (B9FWZ0 /  Os07g0438700) | *O. sativa* | PIRL6 | Plant intracellular Ras-group-related  LRR protein 6 | Q6K7R2 | Os02g0826600 | FunCoup5 | 0.889 | High-confidence functional  coupling (PFC) |  |  |
| OsOXR2 (B9FWZ0 /  Os07g0438700) | *O. sativa* | PANK2 | Pantothenate kinase 2 | Q0J035 | Os09g0533100 | FunCoup5 | 0.888 | High-confidence functional  coupling (PFC) |  |  |
| OsOXR2 (B9FWZ0 /  Os07g0438700) | *O. sativa* | USP | UDP-sugar pyrophosphorylase | Q5Z8Y4 | Os06g0701200 | FunCoup5 | 0.887 | High-confidence functional  coupling (PFC) |  |  |
| OsOXR2 (B9FWZ0 /  Os07g0438700) | *O. sativa* | — | DUF1630 family protein (unknown  function) | Q10NT2 | Os03g0259700 | FunCoup5 | 0.887 | High-confidence functional  coupling (PFC) |  |  |
| OsOXR2 (B9FWZ0 /  Os07g0438700) | *O. sativa* | CoAc1 | Mitochondrial substrate carrier;  Mitochondrial Coenzyme A transporter 1 | Q8S1A3 | Os01g0571000 | FunCoup5 | 0.887 | High-confidence functional  coupling (PFC) |  |  |
| OsOXR2 (B9FWZ0 /  Os07g0438700) | *O. sativa* | — | Uncharacterized ABC1-like kinase  family | A0A0P0W280 | Os03g0698350 | FunCoup5 | 0.886 | High-confidence functional  coupling (PFC) |  |  |
| OsOXR2 (B9FWZ0 /  Os07g0438700) | *O. sativa* | — | Mitochondrial substrate transporter  (Ca2+/metabolite carrier) | Q5NAJ0 | Os01g0708900 | FunCoup5 | 0.886 | High-confidence functional  coupling (PFC) |  |  |
| OsOXR2 (B9FWZ0 /  Os07g0438700) | *O. sativa* | OsMac3 | MAP-kinase activating protein 3 | Q75II8 | Os05g0516900 | FunCoup5 | 0.886 | High-confidence functional  coupling (PFC) |  |  |
| OsOXR2 (B9FWZ0 /  Os07g0438700) | *O. sativa* | — | KH domain-containing protein | Q10KN9 | Os03g0376800 | FunCoup5 | 0.885 | High-confidence functional  coupling (PFC) |  |  |
| OsOXR2 (B9FWZ0 /  Os07g0438700) | *O. sativa* | NTAQ1 | Protein N-terminal glutamine  amidohydrolase | Q60E61 | Os05g0387900 | FunCoup5 | 0.884 | High-confidence functional  coupling (PFC) |  |  |
| OsOXR2 (B9FWZ0 /  Os07g0438700) | *O. sativa* | — | F-box associated type-1 + DUF833  domain protein | Q6K2D5 | Os09g0323500 | FunCoup5 | 0.882 | High-confidence functional  coupling (PFC) |  |  |
| OsOXR2 (B9FWZ0 /  Os07g0438700) | *O. sativa* | KIN14O | Kinesin-like protein KIN-14O | B9G2X9 | Os11g0648100 | FunCoup5 | 0.880 | High-confidence functional  coupling (PFC) |  |  |
| OsOXR2 (B9FWZ0 /  Os07g0438700) | *O. sativa* | OsPGR5 | Proton Gradient Regulation 5 | Q6Z1P2 | Os08g0566600 | STRING v12 | 0.392 | Lower-confidence prediction  (below STRING medium cutoff 0.4) |  | |
| OsOXR2 (B9FWZ0 / Os07g0438700) | *O. sativa* | OsAPC6 | Anaphase-promoting complex subunit 6 | A0A0P0VV58 | Os03g0236966 | STRING v12 | 0.388 | Lower-confidence prediction (below STRING medium cutoff 0.4) |  |  |
| OsOXR2 (B9FWZ0 / Os07g0438700) | *O. sativa* | CRR3 | Probable NAD(P)H dehydrogenase subunit CRR3, chloroplastic | A0A0P0XJZ2 | Os09g0306650 | STRING v12 | 0.388 | Lower-confidence prediction (below STRING medium cutoff 0.4) |  |  |
| OsOXR2 (B9FWZ0 / Os07g0438700) | *O. sativa* | V-ATPase B | Vacuolar proton pump (V-ATPase) subunit B | Q93W07 | Os06g0568200 | STRING v12 | 0.381 | Lower-confidence prediction (below STRING medium cutoff 0.4) |  |  |
| OsOXR2 (B9FWZ0 / Os07g0438700) | *O. sativa* | V-ATPase B | Vacuolar proton pump (V-ATPase) subunit B | Q9ASE0 | Os01g0711000 | STRING v12 | 0.381 | Lower-confidence prediction (below STRING medium cutoff 0.4) |  |  |
| OsOXR2 (B9FWZ0 / Os07g0438700) | *O. sativa* | — | Zinc-finger ZPR1-type domain-containing protein | Q6Z9R0 | Os08g0471900 | STRING v12 | 0.377 | Lower-confidence prediction (below STRING medium cutoff 0.4) |  | |
| OsOXR2 (B9FWZ0 /  Os07g0438700) | *O. sativa* | — | Os07g0543700 protein | A0A0P0X762 | Os07g0543700 | STRING v12 | 0.312 | Lower-confidence prediction  (below STRING medium cutoff 0.4) |  |  |
| OsOXR2 (B9FWZ0 /  Os07g0438700) | *O. sativa* | V-ATPase A | V-type proton ATPase catalytic subunit  A (ID as reported; verify) | AQ651T8 | Os06g0662000 | STRING v12 | 0.312 | Lower-confidence prediction  (below STRING medium cutoff 0.4) |  |  |
| OsOXR2 (B9FWZ0 /  Os07g0438700) | *O. sativa* | V-ATPase A | V-type proton ATPase catalytic subunit  A | Q6EUQ9 | Os02g0175400 | STRING v12 | 0.312 | Lower-confidence prediction  (below STRING medium cutoff 0.4) |  |  |
| OsOXR2 (B9FWZ0 /  Os07g0438700) | *O. sativa* | V-ATPase D | V-type proton ATPase subunit D | Q7XJX6 | Os04g0643100 | STRING v12 | 0.309 | Lower-confidence prediction  (below STRING medium cutoff 0.4) |  |  |
| OsOXR2 (B9FWZ0 /  Os07g0438700) | *O. sativa* | — | No interolog prediction available | — | — | PlaPPISite | — | 0 predicted interactions  returned |  | |
| AtOXR2 (Q9AT60 /  At2g05590) | *A. thaliana* | PP2A B'gamma | Ser/Thr protein phosphatase 2A 59 kDa  regulatory subunit B' gamma | Q8RW96 | At4g15415 | FunCoup5 | 0.915 | High-confidence functional  coupling (PFC) |  | |
| AtOXR2 (Q9AT60 /  At2g05590) | *A. thaliana* | — | At5g40270 (MSN9.18) | Q9FL05 | At5g40270 | FunCoup5 | 0.910 | High-confidence functional  coupling (PFC) |  |  |
| AtOXR2 (Q9AT60 /  At2g05590) | *A. thaliana* | — | N-  acetylglucosaminylphosphatidylinositol deacetylase | Q6DBN0 | At3g58130 | FunCoup5 | 0.908 | High-confidence functional  coupling (PFC) |  |  |
| AtOXR2 (Q9AT60 /  At2g05590) | *A. thaliana* | GTE5 | Transcription factor GTE5, chloroplastic | Q8H1D7 | At1g17790 | FunCoup5 | 0.926 | High-confidence functional  coupling (PFC) |  |  |
| AtOXR2 (Q9AT60 /  At2g05590) | *A. thaliana* | — | Transmembrane protein | F4I861 | At1g57765 | FunCoup5 | 0.905 | High-confidence functional  coupling (PFC) |  |  |
| AtOXR2 (Q9AT60 /  At2g05590) | *A. thaliana* | — | Phosphatidylinositolglycan-like protein | Q8VY53 | At4g35530 | FunCoup5 | 0.908 | High-confidence functional  coupling (PFC) |  |  |
| AtOXR2 (Q9AT60 /  At2g05590) | *A. thaliana* | TG1 | GPCR-type G protein 1 (candidate ABA  receptor) | Q9XIP7 | At1g64990 | FunCoup5 | 0.919 | High-confidence functional  coupling (PFC) |  |  |
| AtOXR2 (Q9AT60 /  At2g05590) | *A. thaliana* | CKB4 | Casein kinase II subunit beta-4 | O80507 | At2g44680 | FunCoup5 | 0.909 | High-confidence functional  coupling (PFC) |  |  |
| AtOXR2 (Q9AT60 /  At2g05590) | *A. thaliana* | ARI7 | Probable E3 ubiquitin-protein ligase  ARI7 | Q84RR0 | At2g31510 | FunCoup5 | 0.907 | High-confidence functional  coupling (PFC) |  |  |
| AtOXR2 (Q9AT60 /  At2g05590) | *A. thaliana* | SSP4b | SCP1-like small phosphatase 4b | F4JQR6 | At4g18140 | FunCoup5 | 0.915 | High-confidence functional  coupling (PFC) |  |  |
| AtOXR2 (Q9AT60 /  At2g05590) | *A. thaliana* | UBP16 | Ubiquitin carboxyl-terminal hydrolase 16 | Q9SB51 | At4g24560 | FunCoup5 | 0.909 | High-confidence functional  coupling (PFC) |  |  |
| AtOXR2 (Q9AT60 /  At2g05590) | *A. thaliana* | UBC36 | Ubiquitin-conjugating enzyme E2 36 | Q9FZ48 | At1g16890 | FunCoup5 | 0.912 | High-confidence functional  coupling (PFC) |  |  |
| AtOXR2 (Q9AT60 /  At2g05590) | *A. thaliana* | — | Eukaryotic aspartyl protease family  protein (MRN17.8) | Q0WRU5 | At5g22850 | FunCoup5 | 0.917 | High-confidence functional  coupling (PFC) |  |  |
| AtOXR2 (Q9AT60 /  At2g05590) | *A. thaliana* | LST8-1 | Target of rapamycin (TOR) complex  subunit LST8-1 | Q9LV27 | At3g18140 | FunCoup5 | 0.910 | High-confidence functional  coupling (PFC) |  |  |
| AtOXR2 (Q9AT60 /  At2g05590) | *A. thaliana* | RTNLB11 | Reticulon-like protein B11 | Q9LT71 | At3g19460 | FunCoup5 | 0.940 | High-confidence functional  coupling (PFC) |  |  |
| AtOXR2 (Q9AT60 /  At2g05590) | *A. thaliana* | PGR5 | Proton Gradient Regulation 5,  chloroplastic | Q9SL05 | At2g05620 | STRING v12 | 0.740 | Prediction |  | |
| AtOXR2 (Q9AT60 /  At2g05590) | *A. thaliana* | CRR3 | Probable NAD(P)H dehydrogenase  subunit CRR3, chloroplastic | Q9ZVE7 | At2g01590 | STRING v12 | 0.718 | Prediction |  |  |
| AtOXR2 (Q9AT60 /  At2g05590) | *A. thaliana* | LIL3.2 | Light-harvesting complex-like protein 3  isotype 2, chloroplastic | Q6NKS4 | At5g47110 | STRING v12 | 0.631 | Prediction |  |  |
| AtOXR2 (Q9AT60 /  At2g05590) | *A. thaliana* | PNSB4 | Photosynthetic NDH subunit of  subcomplex B 4, chloroplastic | Q8RXS1 | At1g18730 | STRING v12 | 0.600 | Prediction |  |  |
| AtOXR2 (Q9AT60 /  At2g05590) | *A. thaliana* | PNSB3 | Photosynthetic NDH subunit of  subcomplex B 3, chloroplastic | Q9LU21 | At3g16250 | STRING v12 | 0.597 | Prediction |  |  |
| AtOXR2 (Q9AT60 /  At2g05590) | *A. thaliana* | PNSB2 | Photosynthetic NDH subunit of  subcomplex B 2, chloroplastic | Q94AQ8 | At1g64770 | STRING v12 | 0.574 | Prediction |  |  |
| AtOXR2 (Q9AT60 /  At2g05590) | *A. thaliana* | SCAR2 | Protein SCAR2 (WAVE complex;  actin/microtubule organization) | Q5XPJ9 | At2g38440 | STRING v12 | 0.572 | Prediction |  |  |
| AtOXR2 (Q9AT60 /  At2g05590) | *A. thaliana* | CRR23/NdhL | NAD(P)H-quinone oxidoreductase  subunit L, chloroplastic | Q9CAC5 | At1g70760 | STRING v12 | 0.497 | Prediction |  |  |
| AtOXR2 (Q9AT60 /  At2g05590) | *A. thaliana* | SCAMP3 | Secretory carrier-associated membrane  protein 3 | Q9M5P2 | At1g61250 | STRING v12 | 0.496 | Prediction |  |  |
| AtOXR2 (Q9AT60 /  At2g05590) | *A. thaliana* | PNSL1 | Photosynthetic NDH subunit of lumenal  location 1, chloroplastic | O80634 | At2g39470 | STRING v12 | 0.440 | Prediction |  |  |
| AtOXR2 (Q9AT60 /  At2g05590) | *A. thaliana* | VHA-C / DET3 | Vacuolar H+-ATPase subunit C (de-  etiolated 3) | Q9SDS7 | At1g12840 | PlaPPISite | 0.36 | Prediction |  | |
